# Serum ^1^H NMR-Based Untargeted Metabolomics for Noninvasive Early Detection of Breast Cancer

**DOI:** 10.1021/acs.jproteome.5c01124

**Published:** 2026-07-15

**Authors:** Akbar Ali, Tengku Ahmad Damitri Al Astani Tengku Din, Maulidiani Maulidiani, Heba Mohammed Arafat, Wan Faiziah Wan Abdul Rahman, Maya Mazuwin Yahya, Suhaini Kadiman, Ras Azira Binti Ramli, Wan Norlina Wan Azman

**Affiliations:** † Department of Chemical Pathology, School of Medical Sciences, Health Campus, 65271 Universiti Sains Malaysia, Kubang Kerian 16150, Kelantan, Malaysia; ‡ Faculty of Science and Marine Environment, 95438 Universiti Malaysia Terengganu, Kuala Nerus 21030, Terengganu, Malaysia; § Department of Pathology, School of Medical Science, Health Campus, Universiti Sains Malaysia, Kubang Kerian 16150, Kelantan, Malaysia; ∥ Department of Surgery, School of Medical Science, Health Campus, Universiti Sains Malaysia, Kubang Kerian 16150, Kelantan, Malaysia, Maya Mazuwin Yahya; ⊥ Anesthesia and Intensive Care Unit, National Heart Institute, Kuala Lumpur 50400, Malaysia; # 65246 Faculty of Medicine Universiti Sultan Zainal Abidin, Kuala Terengganu 20400, Terengganu, Malaysia

**Keywords:** breast cancer, serum metabolomics, ^1^H NMR spectroscopy, machine learning, biomarkers

## Abstract

Breast cancer (BC) is the most common malignancy among women, and late-stage presentation remains common in Asia, highlighting the need for affordable and minimally invasive adjuncts to imaging. This study evaluated whether fasting serum ^1^H NMR metabolomics could distinguish Malaysian women with newly diagnosed treatment-naïve BC from healthy controls and identify perturbed metabolic pathways. After spectral quality control exclusion, 101 participants were analyzed (BC, *n* = 44; control, *n* = 57). PCA and oOPLS-DA showed group separation, and 14 metabolites were deferred significantly after false discovery rate correction. Endogenous metabolites evaluated in BC included 3-hydroxybutyrate, citrate, glutamine, malonate, phenylalanine, dimethyl sulfone, myo-inositol, and L-carnitine, and the levels of formate, tyrosine, histamine, and *p*-hydroxyphenylacetate were reduced. Acetylsalicylate and salicylate were classified as exogenous aspirin-related markers and were excluded from the endogenous biomarker panel. Pathway analysis indicated disruptions in the tricarboxylic acid (TCA) cycle, glyoxylate/dicarboxylate metabolism, butanoate metabolism, and amino acid pathways. Metabolite-based classifiers achieved 0.901 accuracy on an internal held-out test partition (AUC 0.942) and 0.902 ± 0.061 accuracy in repeated stratified 5-fold cross-validation. These internally validated findings define a BC-associated serum metabolomic signature in a Southeast Asian cohort and warrant independent external validation.

## Graphical abstract

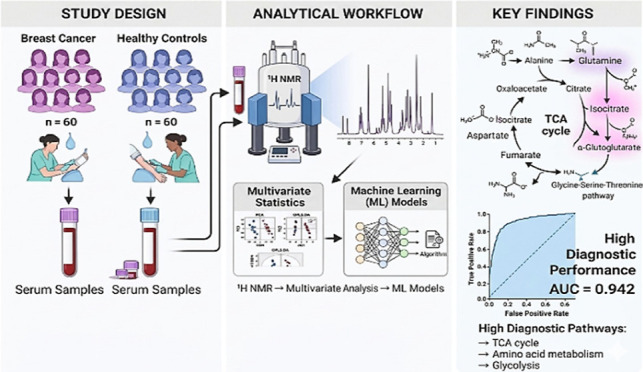

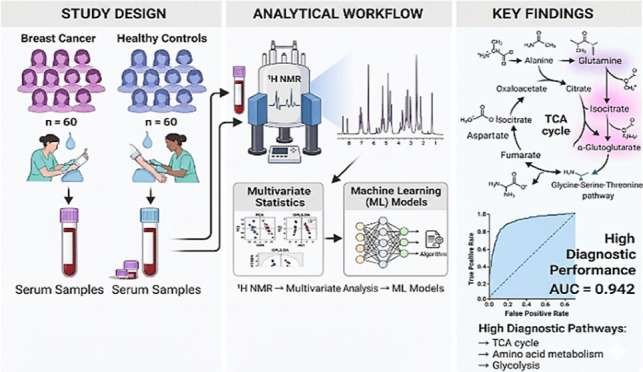

## 1 Introduction

Breast cancer in women is a major global health challenge. In 2022, it accounted for approximately 11.6% of new cancer diagnoses and 6.9% of cancer-related deaths worldwide and is the most commonly diagnosed cancer in women.^1^ In 2020, when breast cancer first surpassed lung cancer in global incidence, there were an estimated 2.3 million new cases and ∼685,000 deaths among women.^2^ Although mortality rates have declined in some parts of the world, the absolute burden continues to rise and is increasingly concentrated in Asia, reflecting demographic changes and evolving risk profiles.^1,2^

Early detection is central to improving patient outcomes. The 2024 guidelines of the United States Preventive Services Task Force (USPSTF) recommend biennial mammography beginning at the age of 40 years for average-risk women.^3,4^ However, current diagnostic and surveillance tools have limitations for early-stage disease. Mammographic performance is attenuated in women with dense breasts and some tumor phenotypes, and definitive diagnosis requires invasive tissue sampling.^3,4^ Major professional guidelines do not recommend the use of serum tumor markers, such as CA 15-3 or CEA, for screening or routine postcurative surveillance due to insufficient sensitivity and specificity, as well as a lack of demonstrated survival benefit; their role is adjunctive, mainly in advanced disease. These constraints underscore the need for minimally invasive biomarkers with improved detection and risk stratification performance.^3,5,6^ Metabolomics, which involves the comprehensive profiling of low-molecular-weight metabolites, provides a system-level readout of phenotypes that integrates genomic, transcriptomic, proteomic, and environmental influences. Small perturbations in enzyme activity can yield amplified changes in metabolite concentrations, enabling the sensitive detection of disease-associated biochemical dysregulation in accessible biofluids.^7–9^ Among the analytical platforms, ^1^H NMR is attractive for clinical translation because it is highly reproducible and inherently quantitative, requires minimal sample preparation, and supports standardized high-throughput workflows suitable for longitudinal and multisite studies.^10–13^ A growing body of work has demonstrated that serum/plasma metabolic profiles can distinguish patients with breast cancer from controls and may inform prognosis and therapeutic response. Case-control and cohort investigations using ^1^H NMR and complementary mass spectrometry have consistently implicated disturbances in amino acid, lipid, and energy metabolism pathways. Machine-learning approaches further enhance the discrimination and prediction of responses to neoadjuvant chemotherapy. Collectively, these findings support metabolomics as a promising approach for noninvasive biomarkers that complement imaging and pathology.^7–9^

Population-specific evaluations are essential because metabolic signatures vary across genetic backgrounds, diets, and environments. In a multiethnic setting with distinctive dietary and environmental exposures, national cancer registry data indicate a high proportion of late-stage (Stage III–IV) presentations (≈48% in 2012–2016), and large-scale molecular profiling has revealed features that differ from those of predominantly European cohorts.^14,15^ Nevertheless, metabolomic investigations of breast cancer in Malaysia remain limited and heterogeneous in terms of biofluid selection and analytical platforms, particularly among newly diagnosed and treatment-naïve patients.^15^

Hypotheses and Objectives: we hypothesized that Malaysian patients with breast cancer would exhibit distinct serum metabolic profiles compared to healthy individuals. Therefore, we conducted an untargeted ^1^H NMR-based serum metabolomics case–control study to characterize the metabolic differences and identify candidate biomarkers that discriminate breast cancer from controls, laying the groundwork for population-tailored, minimally invasive approaches to detection and risk-stratification.

## 2 Materials and Methods

### 2.1 Study Design and Participants

This case-control study was conducted at the Breast Cancer Awareness and Research Unit (BestARi) of Hospital Universiti Sains Malaysia (HUSM) in Kelantan, Malaysia. A total of 120 female participants were enrolled, comprising 60 patients with newly diagnosed breast cancer (BC) and 60 healthy controls (HCs). Following NMR spectral quality control (see Section 2.4), 19 samples did not meet the spectral acceptance criteria and were excluded (BC: *n* = 16; HC: *n* = 3), yielding a final analysis-ready data set of 101 participants (BC: *n* = 44; HC: *n* = 57). Ethical clearance was obtained from the Human Research Ethics Committee of the USM (approval code: USM/JEPeM/KK/23020154), and written informed consent was obtained from all participants prior to enrollment.

Women aged ≥18 years with a recent histopathological diagnosis of primary breast carcinoma were eligible for inclusion in the BC group. Tumor classification followed the American Joint Committee on Cancer (AJCC) TNM staging system^16^ and encompassed cases from stage 0 to stage IV, with tumor categories T1–T4 at initial presentation. To ensure uniformity, only treatment-naïve patients were included; none had undergone surgery, chemotherapy, radiotherapy, endocrine therapy, molecular-targeted therapy, or alternative interventions before sample collection.

The exclusion criteria included conditions or factors that were likely to influence metabolic or immune status, such as current pregnancy, acute or chronic infection within the preceding month, autoimmune or immunosuppressive disorders, diabetes mellitus, and long-term corticosteroid therapy. Patients with a history of alcohol or drug abuse or advanced organ dysfunction (hepatic or renal failure) were also excluded.

The HC group consisted of adult women with no prior history of malignancy, chronic medical conditions, or current breast pathology or lesions. Control participants were recruited from the community and frequency-matched with the BC group by age. The body mass index (BMI) was calculated for all participants as their weight in kilograms divided by the square of their height in meters (kg/m^2^). Medication/supplement use, including aspirin/NSAID use, was not formally recorded at the time of enrollment.

### 2.2 Sample Collection and Storage

Approximately 3 mL of fasting venous blood was collected from each participant in a plain additive-free tube. The samples were allowed to clot at room temperature and centrifuged at 3500 rpm for 10 min to separate the serum. The resulting serum was carefully separated, aliquoted into cryovials, and immediately stored at −80 °C until analysis. All specimens were processed and stored under standardized laboratory conditions to minimize preanalytical variability.

### 2.3 NMR Sample Preparation and Spectral Acquisition

Serum metabolomic profiling was performed by ^1^H NMR spectroscopy. Frozen serum aliquots were shipped on dry ice to the National Institutes of Health (NIH) laboratory for further analysis. Sample preparation followed the protocol described by Abdul-Hamid et al.^9,17^ with minor modifications. Briefly, 200 μL of thawed serum was mixed with 400 μL phosphate buffer in D_2_O (pH 7.4) containing 0.1% (w/v) trimethylsilyl-2,2,3,3-tetradeuteropropionic acid (TSP). TSP served as the internal chemical shift reference (δ 0.00 ppm), and D_2_O provided a deuterium-lock signal. Each 600 μL mixture was transferred into a 5 mm NMR tube (Wilmad, USA), briefly vortexed, and sonicated for 5 min at room temperature to degas and reduce viscosity.

Spectra were acquired on a JEOL 600 MHz NMR spectrometer operating at 600.13 MHz (^1^H frequency) and maintained at 23 °C. For each sample, two spectra were recorded: (i) a standard one-dimensional spectrum with water signal presaturation and (ii) a Carr–Purcell–Meiboom–Gill (CPMG) spin–echo spectrum to attenuate broad macromolecular resonances from proteins and lipoproteins. One-dimensional spectra were collected with 1024 scans, a 90° pulse width of 21.0 μs, a relaxation delay of 2.0 s, and an acquisition time of ∼4 s, yielding ∼8000 data points across a spectral width of −0.3 to 9.8 ppm. CPMG spectra were acquired with 128 scans, a total echo time of 400 ms, a relaxation delay of 5.0 s, and a fixed receiver gain of 90 to ensure consistency. The free induction decays (FIDs) were zero-filled to 64 k points and Fourier-transformed. All spectra were manually phase- and baseline-corrected, and chemical shift calibration was performed using the TSP reference peak (δ = 0.00 ppm). Spectral processing and metabolite identification were conducted using Chenomx NMR Suite version 7.6 (Chenomx Inc., Edmonton, Canada; https://www.chenomx.com/).

### 2.4 Data Preprocessing and Metabolite Identification

All ^1^H NMR spectra were calibrated to the TSP reference signal (δ 0.00 ppm), baseline-corrected, and aligned prior to quantification. The spectral window from δ 0.50 to 10.00 ppm was segmented into fixed-width bins of 0.04 ppm after exclusion of the residual water region (δ 4.70–5.02 ppm), yielding 230 variables for each spectrum.

Spectral preprocessing and quantification were performed using the Chenomx NMR Suite Profiler/Processor (version 7.6; Chenomx Inc., Edmonton, Canada), which was executed at the National Institutes of Health (NIH) using a standardized pipeline with minor manual adjustments to prevent the splitting of key resonances. To mitigate sample-to-sample dilution effects, intensities were normalized using probabilistic quotient normalization (PQN).^18,19^

Metabolite identification was performed from the observed ^1^H NMR spectral peaks using Chenomx based on chemical shifts, multiplicities, and J-coupling patterns. The assignments were subsequently cross-checked against the reference spectra in the Human Metabolome Database (HMDB) and published reports.^20^ Where applicable, the assignments were further corroborated by comparison to authentic standards or spiking experiments. In total, 39 serum metabolites were confidently annotated, including amino acids, organic acids (including ketone bodies), carbohydrates (e.g., glucose), and other small molecules pertinent to breast cancer metabolism (Table 2).

### 2.5 Statistical, Multivariate, and Machine-Learning Analysis

Data distribution was assessed using the Shapiro–Wilk test. Between-group differences (BC vs HC) were evaluated using two-sided Student’s *t* tests for approximately normal variables and Mann–Whitney U tests otherwise (α = 0.05); *p*-values were adjusted using the Benjamini–Hochberg false discovery rate (FDR). Effect sizes are reported as Cohen’s d (parametric) or rank–biserial correlation r (nonparametric).

Multivariate analyses were performed using SIMCA-P+ v14.0 and MetaboAnalyst 5.0.^21^ Principal component analysis (PCA) was used for an exploratory assessment of the data structure and outlier detection. Orthogonal projections to latent structure discriminant analysis (OPLS-DA) was applied as an exploratory discriminant model to identify spectral features driving class separation, including variable importance in projection (VIP) scores and S-plot characteristics; it was not used as the primary diagnostic model because of the known susceptibility of supervised models to overfitting and inflation of Q^2^ in small data sets. Model robustness was evaluated using 200-label permutation testing and cross-validated ANOVA (CV-ANOVA).^22^ Candidate metabolites identified using OPLS-DA (VIP >1.0) were subjected to FDR-corrected univariate testing as an independent confirmation step.

For supervised machine-learning classification, the 101-sample data set was first partitioned into a training set (80%, *n* = 81) and a held-out internal test set (20%, *n* = 20) using stratified random sampling to preserve the class proportions. This partition was fixed before the model fitting. Five classifiers were trained on autoscaled metabolite intensities: L1-regularized logistic regression, support vector machine with a radial basis function kernel, random forest, gradient boosting (XGBoost), and multilayer perceptron.^23^ Hyperparameters were optimized by grid search using stratified 5-fold cross-validation repeated 10 times, which was performed exclusively within the training set. When a class imbalance was present, SMOTE oversampling was applied only within the training fold. The final models were fitted to the full training set and evaluated once by using the locked internal test set. These test set metrics constitute the primary performance estimates reported in Table 4. Model performance was summarized using accuracy, sensitivity (recall), specificity, precision, F1-score, and area under the receiver operating characteristic curve (AUC). As a supplementary internal performance estimate, repeated stratified 5-fold cross-validation (10 repetitions) was performed within the training set, and the results are reported in Supporting Information Table S5. Model interpretability was examined using SHAP (SHapley Additive exPlanations) values to quantify variable-level contributions.^24,25^ Machine-learning analyses were implemented in Python 3.12 (scikit-learn, imbalanced-learn, xgboost, shap, numpy, pandas, and matplotlib). A sensitivity analysis excluding lipoprotein-associated spectral bins was performed by removing 21 of the 230 bins, leaving 209 bins for modeling (Supporting Information Table S9). A separate sensitivity analysis excluding exogenous or pharmaceutical bins was performed by removing 13 of the 230 bins corresponding to acetylsalicylate and salicylate, leaving 217 bins for modeling (Supporting Information Table S10).

### 2.6 Pathway Enrichment

The biological context was derived using MetaboAnalyst 5.0.^26,27^ Enrichment (global test) and topology measurements were applied to the discriminatory metabolites. Pathways with a Holm-adjusted *p*-value ≤0.05 were considered significantly perturbed in BC compared to HC.

## 3 Results

### 3.1 Participant Characteristics

We analyzed 101 women who passed spectral quality control (BC, *n* = 44; HC, *n* = 57). Chronological age did not differ between the groups (BC: 44 [37–50] years; HC: 43 [38–54] years; *p* >0.05; Table 1). Reproductive and anthropometric variables were also comparable: age at menarche (12 [12–14] vs 12 [11–13] years), parity (2 [1–2] vs 2 [1–2]), postmenopausal status (20.2% vs 30.3%), and body mass index (BMI; 25.07 [22.72–28.36] vs 24.02 [22.20–28.13] kg/m^2^) (all *p* >0.05; Table 1).

**Table 1:** Baseline Characteristics of the Study Population (HC vs BC)

characteristic	control (*n* = 57)	breast cancer (*n* = 44)	p-value
age (years)	43 (38–54)	44 (37–50)	>0.05
no. of children	2 (1–2)	2 (1–2)	>0.05
age at menarche (years)	12 (11–13)	12 (12–14)	>0.05
menopausal (yes, %)	30.3	20.2	>0.05
BMI (kg/m^2^)	25.07 (22.72–28.36)	24.02 (22.20–28.13)	>0.05
total cholesterol (mg/dL)	194.18 ± 28.31	208.06 ± 31.40	0.002
ApoA1 (mg/dL)	147.22 ± 28.66	147.64 ± 27.34	>0.05
ApoB100 (mg/dL)	91 (78–104)	101 (85–118)	0.008
triglycerides (mg/dL)	76.55 (56.83–98.74)	86.73 (67.69–120.16)	0.004
FABP4 (ng/mL)	13.09 (8.73–18.05)	17.53 (13.15–23.33)	<0.001
FABP5 (ng/mL)	6.12 (5.44–7.91)	7.00 (5.26–9.04)	>0.05
CETP activity (pmol/μL)	47.87 (26.95–69.54)	51.98 (31.71–76.43)	>0.05
CETP concentration (ng/μL)	596.51 ± 163.39	579.63 ± 155.76	>0.05
soluble CD36 (pg/mL)	908.67(762.00–1037.56)	897.00(763.37–997.56)	>0.05

#### 3.1.1 Biochemical Parameters

Several lipid-related biomarkers were higher in the BC group than in the HC group. Total cholesterol increased (208.06 ± 31.40 vs 194.18 ± 28.31 mg/dL; *p* = 0.002), apolipoprotein B100 (ApoB100) was higher (101 [85–118] vs 91 [78–104] mg/dL; *p* = 0.008), and triglycerides were elevated (86.73 [67.69–120.16] vs 76.55 [56.83–98.74] mg/dL; *p* = 0.004).

In contrast, apolipoprotein A1 (ApoA1) levels did not differ significantly between the groups (147.64 ± 27.34 vs 147.22 ± 28.66 mg/dL; *p* >0.05). Among the adipokines, fatty acid-binding protein 4 (FABP4) was higher in BC (17.53 [13.15–23.33] vs 13.09 [8.73–18.05] ng/mL; *p* <0.001), whereas FABP5, cholesteryl ester transfer protein (CETP) activity and concentration, and soluble CD36 showed no statistically significant between-group differences (all *p* >0.05; Table 1).

Data were obtained from 101 women (BC, *n* = 44; HC, *n* = 57). Continuous variables were summarized as mean ± standard deviation (SD) when approximately normal and as median [interquartile range, IQR] when non-normal. Categorical variables are expressed as percentages. Between-group differences were assessed using two-sided Student’s *t* tests (parametric data), Mann–Whitney U tests (nonparametric data), or χ^2^ tests for categorical data, as appropriate. Exact *p*-values are reported where available; otherwise, *p* >0.05 indicates nonsignificance. Unless otherwise stated, no adjustments were applied for multiple comparisons (α = 0.05).

### 3.2 NMR Spectral Profiles and Metabolite Identification

Representative one-dimensional ^1^H CPMG NMR spectra of serum samples from BC and HC participants are shown (Figure 1A–C). The Carr–Purcell–Meiboom–Gill (CPMG) sequence was applied to suppress broad macromolecular resonances and enhance signals from low-molecular-weight metabolites.^28^ Figure 1A presents the full spectral window (0–10 ppm) for the representative BC and HC samples, illustrating their distinct metabolic profiles. Targeted expansions of the aliphatic region (0.5–3.0 ppm; Figure 1B) and the 3.0–5.5 ppm region, which includes carbohydrate and selected amino acid resonances (Figure 1C), highlight the qualitative between-group differences.

**Figure 1:**
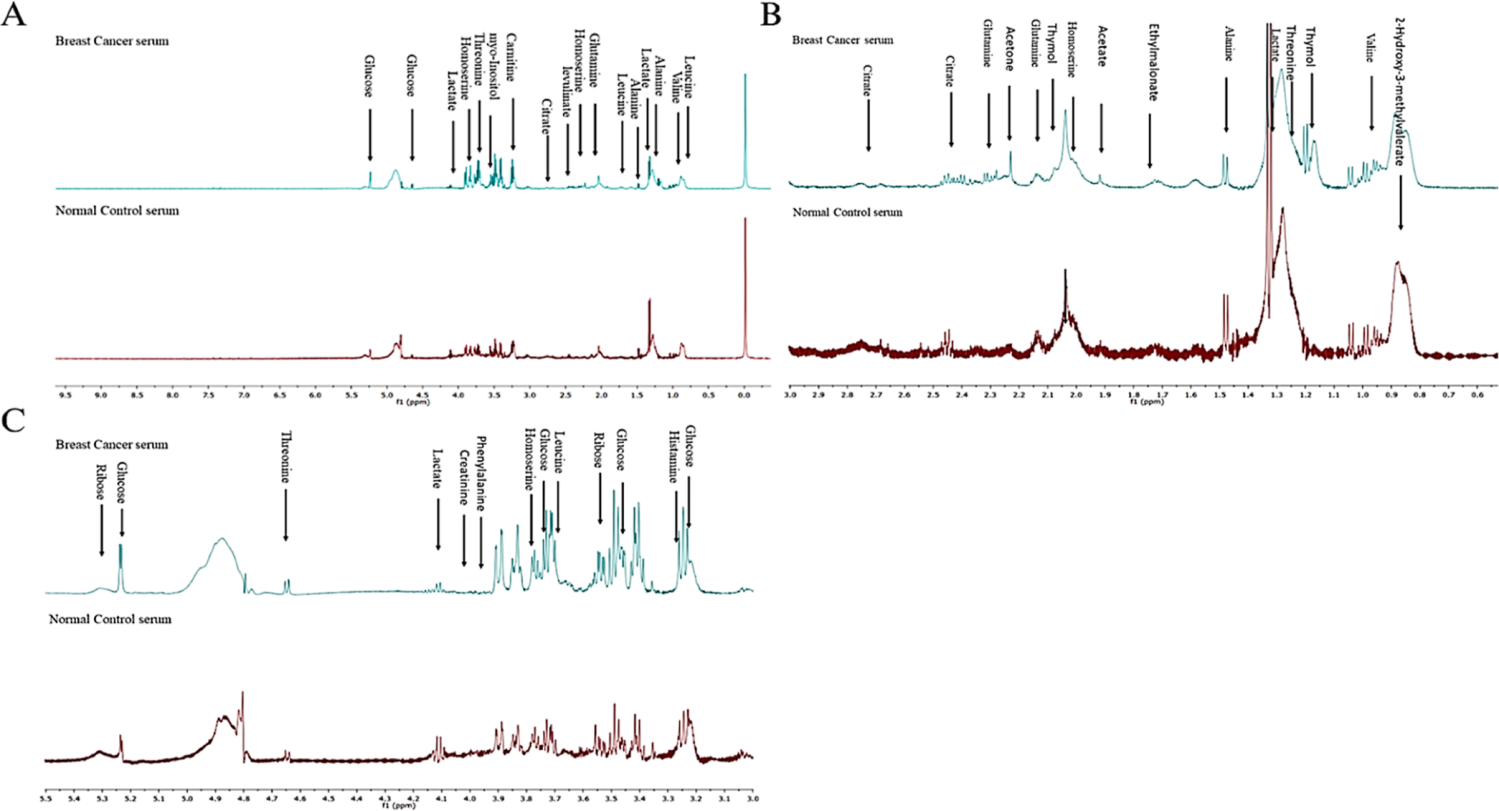
Representative ^1^H CPMG NMR serum spectra in breast cancer and healthy controls.

Visual inspection indicated relatively higher signals in BC for lactate (doublet ∼1.33 ppm; quartet ∼4.11 ppm), 3-hydroxybutyrate (doublet ∼1.20 ppm), branched-chain amino acids (valine/leucine/isoleucine multiplets ∼0.9–1.0 ppm), and glucose (anomeric and ring-proton resonances ∼5.2 and 3.2–4.0 ppm, respectively). Metabolite assignments were established by matching chemical shifts, multiplicities, and J-coupling patterns to reference spectra in the Chenomx NMR Suite and cross-checking against the Human Metabolome Database (HMDB).^20^ In total, 39 metabolites were annotated with high confidence and are listed in Table 2, together with their characteristic chemical shift positions.

**Table 2:** Metabolites Annotated from Serum 1D ^1^H CPMG NMR Spectra

no	HMDB ID	metabolite	characteristic ^1^H NMR signals, δ (ppm) (multiplicity)	chemical class	principal amino acid link/precursor	principal metabolic pathway
1	HMDB0001659	acetone	2.23 (s)	ketone body	leucine/lysine linked; also, fatty-acid derived	ketone body metabolism
2	HMDB0000128	acetate	1.92 (s)	short-chain carboxylic acid	no primary amino acid precursor assigned	acetyl-CoA/energy metabolism
3	HMDB0000060	acetoacetate	2.28 (s), 3.43 (s)	ketone body	leucine/lysine linked; also, fatty-acid derived	ketone body metabolism
4	HMDB0000062	L-carnitine	3.23 (s)	quaternary ammonium compound	lysine and methionine linked	fatty-acid transport and β-oxidation
5	HMDB0000064	creatine	3.03 (s), 3.93 (s)	guanidino compound	glycine, arginine, and methionine linked	creatine metabolism
6	HMDB0000094	citrate	2.70 (d, *J* = 16.2 Hz), 2.50 (d, *J* = 16.2 Hz)	tricarboxylic acid	no primary amino acid precursor assigned	TCA cycle
7	HMDB0000123	glycine	3.56 (s)	amino acid	glycine/serine interconversion	glycine, serine, and one-carbon metabolism
8	HMDB0000142	formate	8.46 (s)	one-carbon metabolite	serine/glycine linked	folate-mediated one-carbon metabolism
9	HMDB0000148	glutamate	2.33 (m), 2.14 (m), 2.05 (m)	amino acid	glutamine/proline/arginine linked	alanine, aspartate, and glutamate metabolism
10	HMDB0000158	tyrosine	7.20 (d), 6.90 (d), 3.90 (m), 3.00 (m)	aromatic amino acid	phenylalanine derived	phenylalanine and tyrosine metabolism
11	HMDB0000159	l-phenylalanine	7.32 (d), 4.00 (t), 3.10 (m, overlap)	aromatic amino acid	essential amino acid	phenylalanine metabolism
12	HMDB0000161	l-alanine	1.48 (d), 3.78 (m)	amino acid	pyruvate linked	alanine metabolism/pyruvate metabolism
13	HMDB0000167	l-threonine	3.60 (d), 4.32 (t, overlap)	amino acid	essential amino acid	glycine, serine, and threonine metabolism
14	HMDB0000187	serine	3.80 (s), 4.00 (d, overlap)	amino acid	3-phosphoglycerate linked	serine and one-carbon metabolism
15	HMDB0000719	homoserine	2.05 (m), 2.15 (m), 3.77 (m), 3.85 (m, overlap)	nonproteinogenic amino acid	aspartate family linked	aspartate-family amino acid metabolism
16	HMDB0000122	d-glucose	5.26 (s), 4.00 (s), 3.90 (d), 3.85 (m), 3.80 (m), 3.72 (t), 3.70 (m), 3.48 (t), 3.40 (t), 3.23 (m)	hexose carbohydrate	no primary amino acid precursor assigned	glycolysis/gluconeogenesis
17	HMDB0000190	lactate	1.33 (d), 4.11 (m)	α-hydroxy acid	alanine/pyruvate linked	glycolysis/pyruvate metabolism
18	HMDB0000214	ornithine	3.77 (t), 3.04 (m), 1.92 (s), 1.72 (s)	nonproteinogenic amino acid	arginine/glutamate linked	urea cycle/arginine metabolism
19	HMDB0000243	pyruvate	2.37 (s)	α-keto acid	alanine, serine, cysteine, and threonine linked	glycolysis/pyruvate metabolism
20	HMDB0000011	3-hydroxybutyrate	1.20 (d), 2.30 (m), 2.41 (m)	ketone body	leucine/lysine linked; also fatty-acid derived	ketone body metabolism
21	HMDB0000254	succinate	2.41 (s)	dicarboxylic acid	valine, isoleucine, methionine, and threonine linked	TCA cycle
22	HMDB0000562	creatinine	3.03 (s), 4.05 (s)	cyclic amide	creatine derived	creatine metabolism
23	HMDB0000641	glutamine	3.77 (t), 2.46 (t, overlap), 2.44 (t, overlap), 2.14 (m), 2.12 (m, overlap)	amino acid amide	glutamate linked	glutamine–glutamate/nitrogen metabolism
24	HMDB0000687	leucine	3.72 (t), 1.74 (m, overlap), 1.72 (m, overlap), 1.70 (m, overlap), 0.96 (d), 0.95 (d)	branched-chain amino acid	essential amino acid	branched-chain amino acid metabolism
25	HMDB0000691	malonate	3.12 (s)	dicarboxylic acid	no primary amino acid precursor assigned	dicarboxylate metabolism
26	HMDB0000883	valine	3.61 (s), 1.04 (d), 0.99 (d)	branched-chain amino acid	essential amino acid	branched-chain amino acid metabolism
27	HMDB0004983	dimethyl sulfone	3.15 (s)	organosulfur compound	methionine/cysteine linked; possible exogenous contribution	sulfur metabolism/oxidative stress-associated metabolism
28	HMDB0003243	acetoin	2.23 (s)	hydroxy ketone	no primary amino acid precursor assigned	pyruvate-related metabolism
29	HMDB0000043	betaine	3.89 (s), 3.24 (s)	trimethylated zwitterion	choline/glycine linked	choline oxidation/one-carbon metabolism
30	HMDB0000211	myo-inositol	4.06 (s), 3.61 (m), 3.44 (dd), 3.25 (t)	cyclitol/polyol	no primary amino acid precursor assigned	inositol-related metabolism
31	HMDB0000086	glycerophosphorylcholine	3.67 (d, overlap), 3.66 (s), 3.23 (s)	choline phospholipid derivative	choline linked	glycerophospholipid metabolism
32	HMDB0000259	serotonin	7.42 (d), 7.08 (s)	biogenic amine	tryptophan derived	tryptophan metabolism
33	HMDB0001879	acetylsalicylate	7.38 (t), 7.14 (d), 2.32 (s)	aromatic acetylated salicylate	exogenous	xenobiotic/drug metabolism
34	HMDB0000283	D-ribose	5.26 (s), 4.00 (s), 3.82 (s), 3.70 (s), 3.67 (t), 3.60 (d)	pentose carbohydrate	no primary amino acid precursor assigned	pentose phosphate pathway/nucleotide metabolism
35	HMDB0000870	histamine	7.85 (s), 7.08 (s), 3.02 (t)	biogenic amine	histidine derived	histidine metabolism
36	HMDB0000622	ethylmalonate	1.71 (t), 0.88 (m, overlap)	dicarboxylic acid derivative	no primary amino acid precursor assigned	fatty-acid/dicarboxylate metabolism
37	HMDB0000720	levulinate	2.76 (s), 2.39 (t), 2.23 (s)	keto acid derivative	no primary amino acid precursor assigned	intermediary carbon metabolism
38	HMDB0000317	2-hydroxy-3-methylvalerate	1.72 (s), 0.95 (d), 0.86 (t, overlap)	branched hydroxy acid	isoleucine linked	branched-chain amino acid metabolism
39	HMDB0001875	methanol	3.34 (s)	one-carbon alcohol	no primary amino acid precursor assigned	xenobiotic/microbial-associated metabolism

As peak-wise visual comparisons are limited by resonance overlap and interindividual variability, univariate inspection alone did not reliably distinguish BC from HC patients. Accordingly, multivariate statistical analysis, including OPLS-DA, was performed to achieve group separation and to identify metabolites contributing most strongly to the observed differences (see Methods).

Figure 1 shows a full spectral window (0–10 ppm) for representative BC and HC serum samples, illustrating the overall metabolic fingerprints. Figure 1 shows an aliphatic region (0.5–3.0 ppm) highlighting signals from branched-chain amino acids (valine, leucine/isoleucine), alanine, lactate, citrate, acetate, and other related metabolites. Figure 1 shows the region spanning 3.0–5.5 ppm, highlighting resonances from glucose (anomeric and ring protons), myo-inositol, creatine/creatinine, threonine, and phenylalanine/tyrosine (where resolved). The spectra were vertically offset for clarity, and the arrows denote the principal resonances used for the assignment. Metabolite assignments were guided by chemical shifts (δ, ppm), multiplicity, and J-coupling patterns and cross-checked against reference libraries (for example, Chenomx) and the Human Metabolome Database (HMDB) (https://www.hmdb.ca/). Some of the labeled peaks represent overlapping resonances. BC, breast cancer; HCs, healthy controls; CPMG, Carr–Purcell–Meiboom–Gill (see Table 2 for a full list of annotated metabolites and characteristic chemical shifts).

Table 2 provides the metabolites annotated from the serum 1D ^1^H CPMG NMR spectra with the molecular framework/chemical class, principal amino acid link/precursor, and principal metabolic pathway.

### 3.3 Multivariate Analysis and Identification of Candidate Biomarkers

PCA of autoscaled metabolite data captured the major sources of variance in PC1 and PC2 and showed only partial separation between BC and HC samples (Figure 2A). This incomplete separation motivated supervised discriminant modeling. OPLS-DA was therefore fitted to the quantified metabolites after mean centering and scaling (see Methods). The final OPLS-DA model (M3) contained one predictive and one orthogonal component and achieved *R* ^2^X­(cum) = 0.655, *R* ^2^Y­(cum) = 0.851, and *Q* ^2^(cum) = 0.722 (Table 3). In the score plot, BC and HC were separated primarily along the first predictive component, with limited overlap indicating systematic metabolic differences.

**Figure 2:**
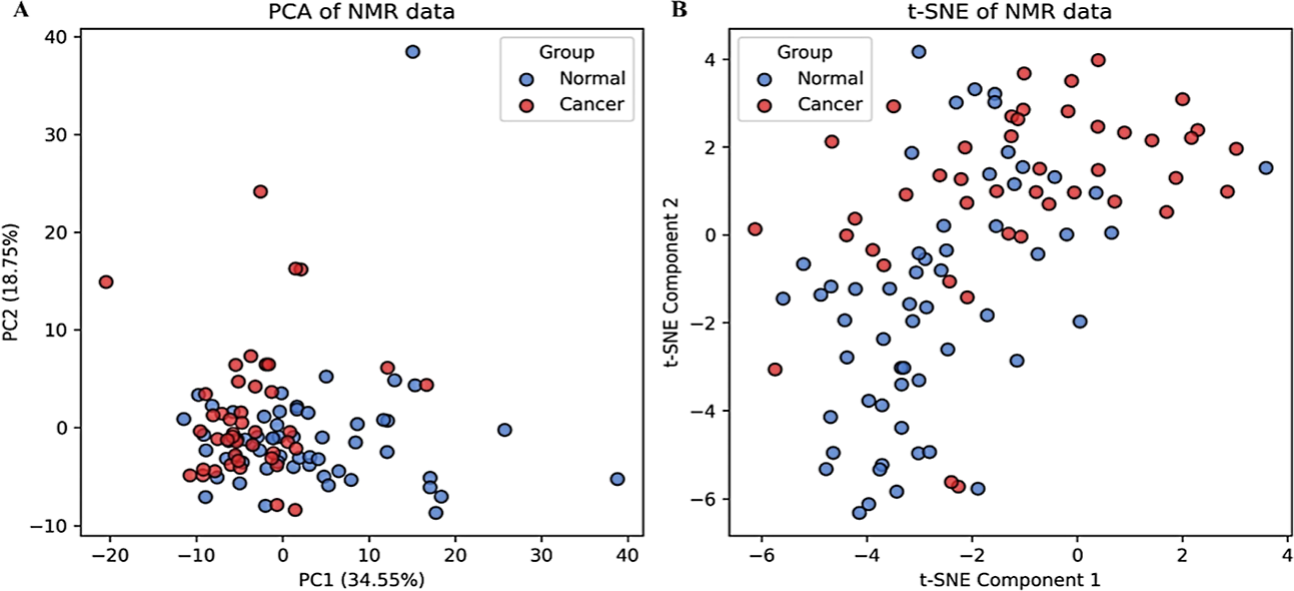
Unsupervised visualization of serum ^1^H NMR metabolomic profiles.

**Table 3:** OPLS-DA Performance Metrics for Distinguishing BC from HC

groups	*R* ^2^_X_ (cum)	*R* ^2^Y (cum)	*Q* ^2^ (cum)
OPLS-DA (1 predictive +1 orthogonal; M3)	0.655	0.851	0.722

Model robustness was supported by 200-label permutation testing, in which the observed *Q* ^2^ exceeded that of all permuted models (Supporting Information Figures S1 and S2) and cross-validated ANOVA (CV-ANOVA, *p* <0.0001; Supporting Information Table S1). Loading inspection using the OPLS-DA S-plot (covariance p vs correlation p; Figure 4) highlighted the variables that contributed most strongly to class discrimination. The metabolites that were relatively elevated in BC included acetate, citrate, glycine, formate, glutamate, alanine, homoserine, glucose, pyruvate, 3-hydroxybutyrate, glutamine, malonate, valine, myo-inositol, acetylsalicylate, levulinate, 2-hydroxy-3-methylvalerate, and methanol. Methanol is likely to be an exogenous compound and should be interpreted cautiously. The metabolites that were relatively higher in the HC group included D-ribose, histamine, ethylmalonate, creatine, lactate, and leucine. These directions were further examined using FDR-adjusted univariate tests (see Methods).

Together, the PCA, OPLS-DA, permutation testing, and CV-ANOVA results supported a reproducible metabolic distinction between BC and HC under the specified preprocessing and validation schemes.

The explained cumulative variance and predictability are reported as *R* ^2^X, *R* ^2^Y, and *Q* ^2^, respectively. The final model contained one predictive component and one orthogonal component. Higher *R* ^2^X, *R* ^2^Y, and *Q* ^2^ values indicate better fit and predictive abilities. Data were mean-centered and scaled before modeling (see Methods). Model robustness was evaluated using permutation testing and cross-validated ANOVA.

#### 3.3.1 Identification of Candidate Biomarkers

PCA showed that PC1 and PC2 explained 34.55% and 18.75% of the variance, respectively, and revealed only partial separation between the BC and HC samples (Figure 2A). Complementary *t*-distributed stochastic neighbor embedding (*t*-SNE) visualization indicated a moderate class structure with a residual overlap (Figure 2B). These unsupervised findings motivated the use of supervised modeling with OPLS-DA to identify discriminant metabolites and prioritize candidate biomarkers based on loading, S-plots, and VIP scores, followed by univariate confirmation.

Figure 2 shows the PCA score plot of autoscaled binned spectral data colored by class (blue = normal, red = cancer). The axes show the proportion of variance explained by PC1 (34.55%) and PC2 (18.75%), indicating only a partial separation between the classes. Figure 2 shows the t-SNE of the same data set, which indicates a moderate class structure with residual overlap.

Supervised discrimination was evaluated by using PLS-DA and OPLS-DA. The PLS-DA score plot (Figure 3A) shows the separation between classes (orange = normal; blue = cancer), with 95% confidence ellipses indicating within-group dispersion. In the sensitivity analysis, the four-component model explained and achieved *R* ^2^X­(cum) = 0.655, *R* ^2^Y­(cum) = 0.851, and *Q* ^2^(cum) = 0.722, and a 200-permutation test indicated that performance was unlikely to be a chance (*p* = 0.005).

**Figure 3:**
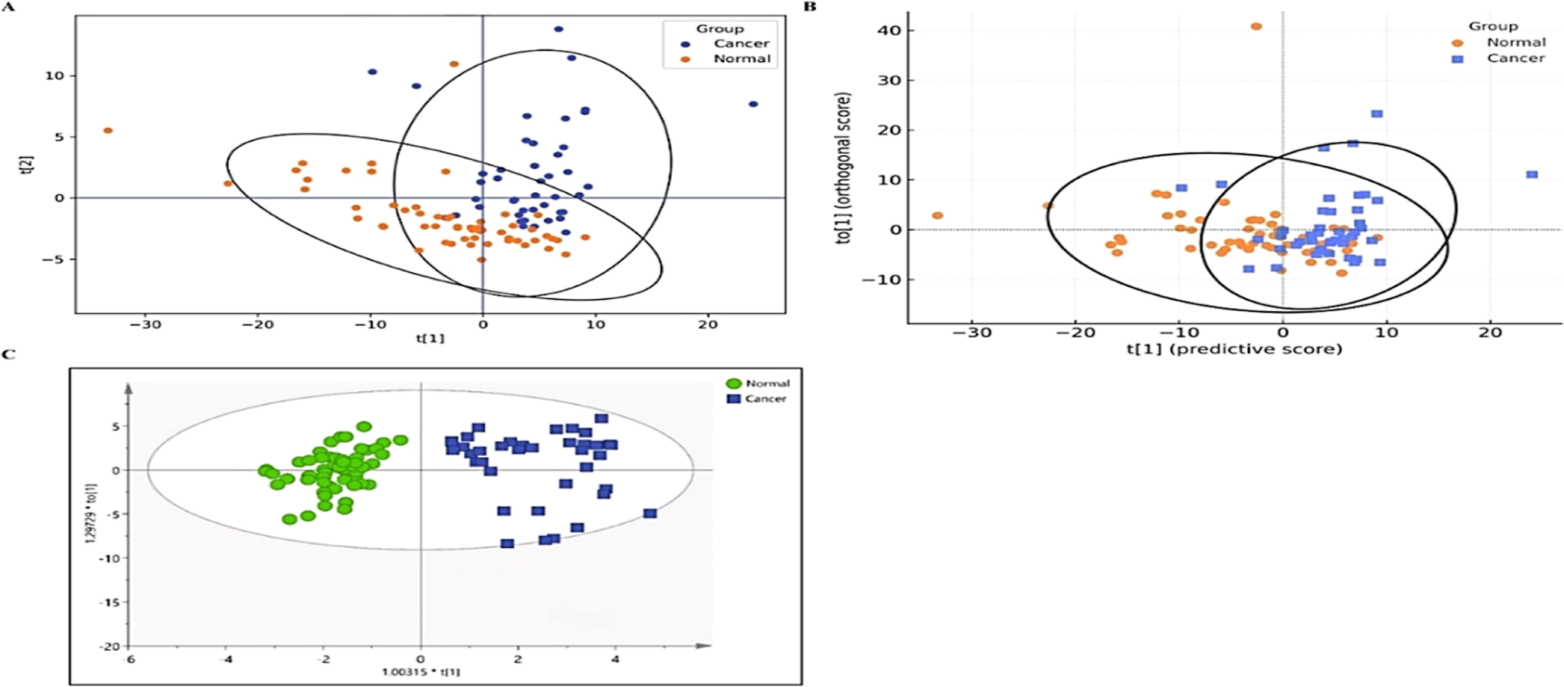
Supervised discrimination of serum ^1^H NMR metabolomic profiles.

To identify the drivers of discrimination, we inspected the OPLS-DA S-plot score plot in generated SIMCA (Figure 4A), which displays covariance p[1] versus correlation p­(corr)[1] for each metabolite. Metabolites relatively elevated in the cancer group included acetate, citrate, glycine, formate, glutamate, alanine, homoserine, glucose, pyruvate, 3-hydroxybutyrate, glutamine, malonate, valine, and myo-inositol, with additional signals for acetylsalicylate, levulinate, and 2-hydroxy-3-methylvalerate. Methanol was also detected and was likely exogenous; therefore, it should be interpreted with caution. Features that were relatively higher in the normal group included d-ribose, histamine, ethylmalonate, creatine, lactate, and leucine. The VIP scores supported these patterns (VIP ≥1.0; Supporting Information Table S2), indicating a coordinated metabolic signature rather than a single dominant biomarker.

**Figure 4:**
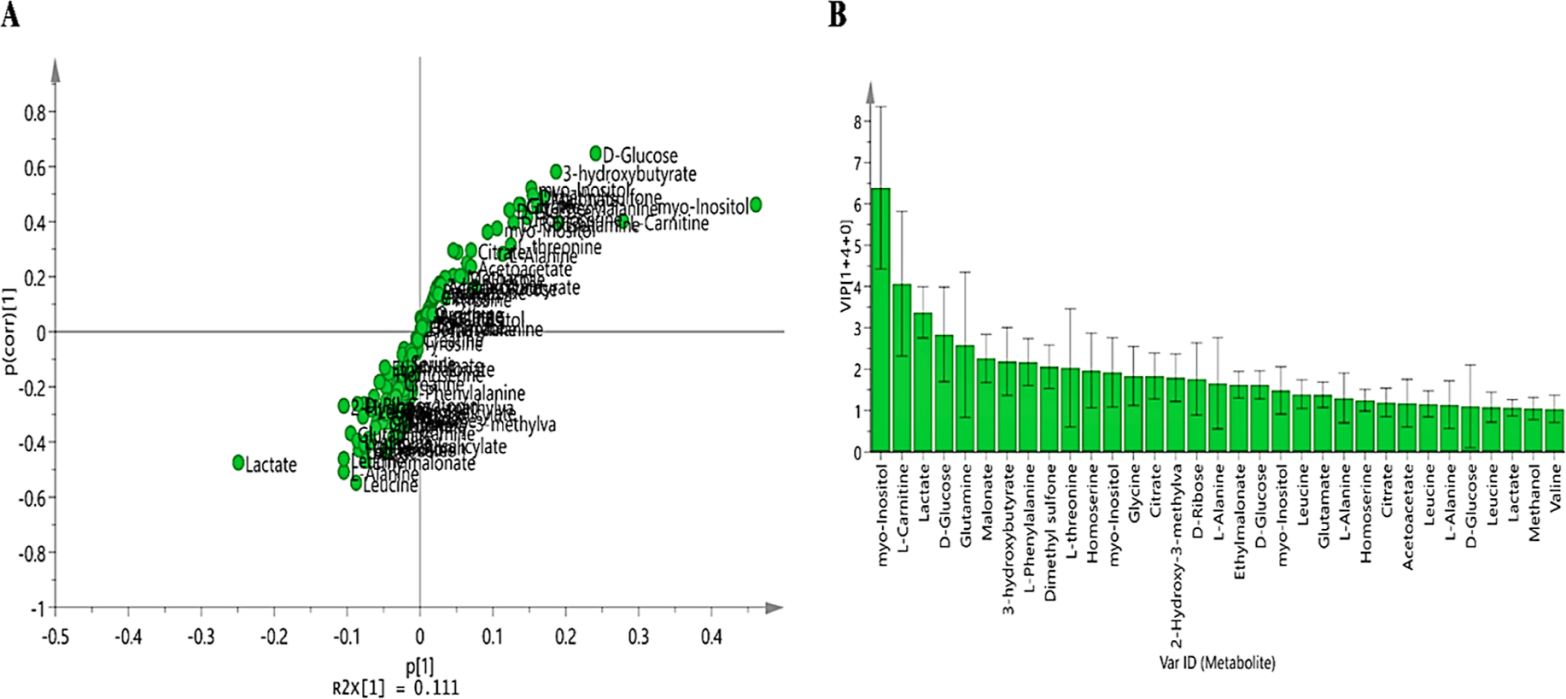
Discriminant metabolites from OPLS-DA of serum profiles.

As a sensitivity check, we approximated the predictive subspace of OPLS-DA by evaluating the PLS-DA on the binned spectra. The four-component model explained *R* ^2^X = 0.600 and *R* ^2^Y = 0.736, yielding a *Q* ^2^ value of 0.269. In stratified 5-fold cross-validation, logistic regression classifiers trained on fold-wise partial least-square (PLS) scores achieved an accuracy of 0.851 and an AUROC of 0.915. A 200-permutation test yielded a *p*-value of 0.005, indicating statistical significance. These findings are directionally consistent with the OPLS-DA results, although with a lower cross-validated *Q* ^2^ (Supporting Information Figure S3 and Table S3).

Figure 3 shows the PLS-DA score plot t[1] vs t[2] of autoscaled binned spectra, colored by class (orange = normal, blue = cancer); ellipses denote 95% covariance regions. The model diagnostics and cross-validated performance are reported in the Methods and Supporting Information (Figures S3–S5; Table S4). Figure 3 shows the OPLS-DA score plot generated in Python showing t[1] (predictive) vs t[1] (orthogonal). Summary statistics and validation (permutation; CV-ANOVA) are presented in Table 3 and the Supporting Information. Figure S3 shows the OPLS-DA score plot produced in SIMCA, t[1] vs to[1]; colors follow the SIMCA export (green = normal, blue = cancer). The separation patterns in (B) and (C) were consistent, providing cross-software confirmation. Abbreviations: PLS-DA, partial least-square discriminant analysis; OPLS-DA, orthogonal projections to latent structure-discriminant analysis.

#### 3.3.2 Metabolites Contributing to Class Separation (VIP)

The OPLS-DA loadings (S-plot; covariance p[1] vs correlation p­(corr)[1]) highlighted the metabolites driving class discrimination. Signals that were relatively higher in the cancer group included acetate, citrate, glycine, formate, glutamate, alanine, homoserine, glucose, pyruvate, 3-hydroxybutyrate, glutamine, malonate, valine, and myo-inositol. Additional peaks were observed for acetylsalicylate, levulinate, and 2-hydroxy-3-methylvalerate. Features relatively higher in the normal group included D-ribose, histamine, ethylmalonate, creatine, lactate, and leucine. Variable-importance scores from the OPLS-DA model corroborated these patterns: 31/39 annotated metabolites had VIP ≥1.0 (VIP–CV-SE >1.0; Supporting Information Table S2).

Unsupervised hierarchical clustering of autoscaled serum metabolite concentrations (row-wise *z*-scores, Euclidean distance, Ward linkage) revealed two principal sample branches that largely corresponded to the BC and HC groups (Figure 5). Columns represent individual subjects (annotation bar: orange = normal, blue = cancer), and rows represent 39 annotated metabolites (labeled by name or representative ppm where applicable). BC samples showed broadly higher relative abundances (warm tones) than those of HC, with a smaller subset of metabolites being higher in HC (cool tones). For example, 3-hydroxybutyrate, glutamine, malonate, and myoinositol levels are elevated in BC. In contrast, formate, histamine, creatine, and selected branched-chain amino acids were relatively higher in the HC. This structure is consistent with the multivariate discrimination reported above (PCA/OPLS-DA) and indicates that group differences reflect coordinated shifts across multiple metabolites. Covarying metabolite clusters were also apparent, suggesting pathway-level perturbations in BC. A complementary panel restricted to the top VIP metabolites from the OPLS-DA model reproduced the same sample grouping, underscoring the fact that the separation was driven by biologically coherent features rather than noise.

**Figure 5:**
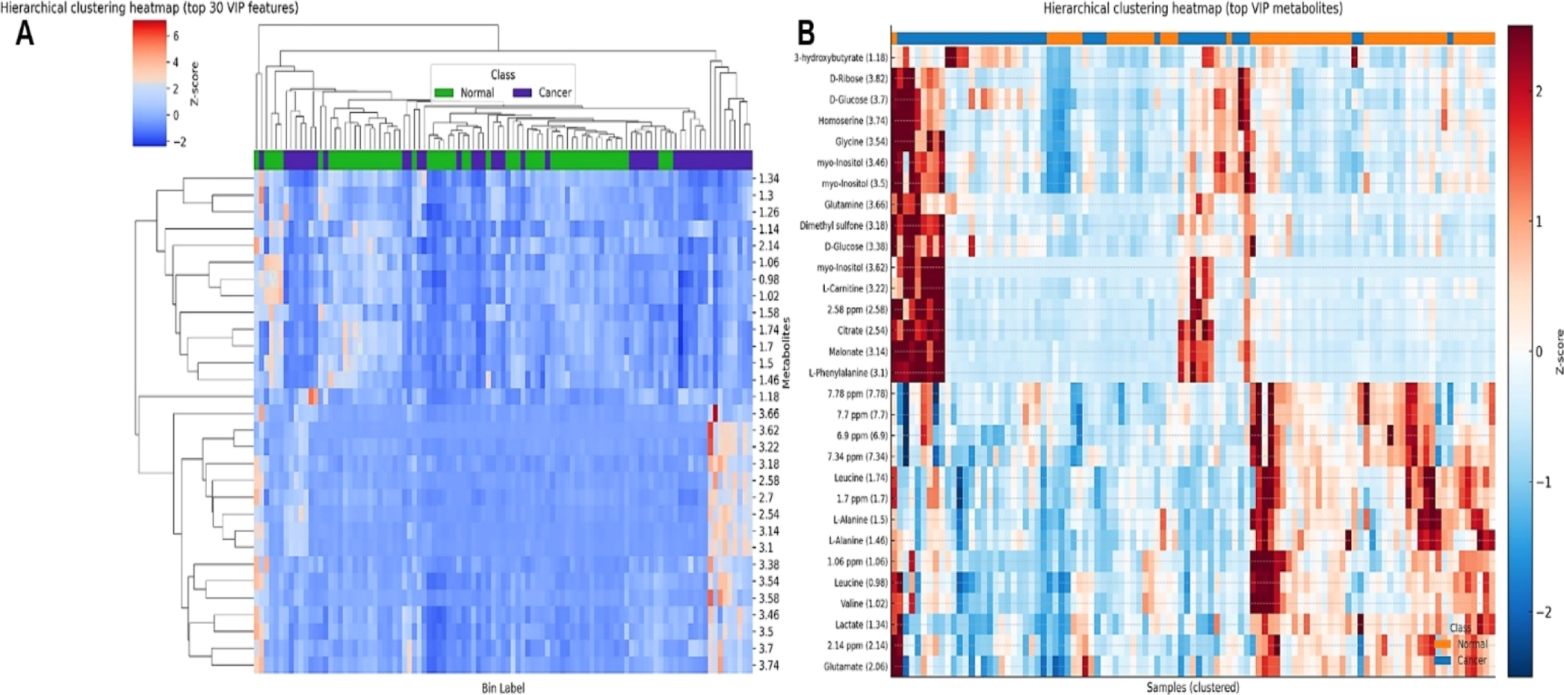
Heatmap of metabolite levels and unsupervised hierarchical clustering of samples.

Figure 5 shows the heatmap of autoscaled metabolite intensities (row-wise *z*-scores; blue = low, red = high) across individual samples. Columns represent subjects (annotation bar: orange = normal, blue = cancer); rows represent identified metabolites (ppm in parentheses). The samples were hierarchically clustered (Euclidean distance and Ward linkage), showing the separation of most cancers from normal samples. Row clusters indicated coordinated changes; for example, 3-hydroxybutyrate, glutamine, malonate, and myo-inositol levels tended to be higher in cancer patients, whereas formate, histamine, and creatine levels were relatively higher in healthy individuals. Figure 5 shows the heatmap restricted to the top VIP metabolites from the OPLS-DA model (same scaling and legend), illustrating a concordant class structure with a tighter within-group similarity.

### 3.4 Machine-Learning Classification

To evaluate the diagnostic utility of the serum metabolite panel, five supervised classifiers were trained on the autoscaled features: L1-penalized logistic regression (LR-L1), support vector machine with a radial basis function kernel (SVM-RBF), random forest (RF), gradient boosting (XGBoost), and one hidden layer multilayer perceptron (MLP). Hyperparameters were optimized using a grid search with stratified 5-fold cross-validation repeated 10 times within the training set. When a class imbalance arose, SMOTE was applied within the training folds to prevent information leakage (see Methods). The positive class was defined as the BC. The performance was summarized by the accuracy, sensitivity (recall), specificity, precision, F1-score, and area under the receiver operating characteristic curve (AUC). Model interpretability was examined by using SHAP values.

For the prespecified primary analysis restricted to metabolites, the data set was partitioned into a training set (80%, *n* = 81) and a locked internal held-out test partition (20%, *n* = 20) before model fitting. For the internal held-out test partition, LR-L1 achieved the best overall performance (accuracy 0.901, AUC 0.942), followed by SVM-RBF (accuracy 0.832, AUC 0.940), RF (accuracy 0.810, AUC 0.935), and MLP (accuracy 0.810, AUC 0.940), whereas XGBoost performed less well (accuracy 0.620, AUC 0.860) (Table 4; ROC curves are shown in Figure 6). Repeated stratified 5-fold cross-validation yielded more conservative internal estimates, with LR-L1 again showing the strongest overall performance (accuracy 0.902 ± 0.061, AUC 0.963 ± 0.032), followed by RF (accuracy 0.826 ± 0.086, AUC 0.926 ± 0.063), MLP (accuracy 0.805 ± 0.097, AUC 0.903 ± 0.075), SVM-RBF (accuracy 0.780 ± 0.095, AUC 0.900 ± 0.087), and XGBoost (accuracy 0.765 ± 0.105, AUC 0.851 ± 0.088) (Table 5). The confusion matrices indicated a relatively balanced sensitivity and specificity for LR-L1, with weaker classification performance for the other models (Supporting Information Figure S5). The tuned hyperparameters are listed in Supporting Information Table S4.

**Table 4:** Hold-Out Test Performance (Metabolites Only)

model	accuracy	AUC
logistic regression (L1)	0.901	0.942
SVM (RBF kernel)	0.832	0.940
random forest	0.810	0.935
MLP (1 × 100)	0.810	0.940
XGBoost	0.620	0.860

**Figure 6:**
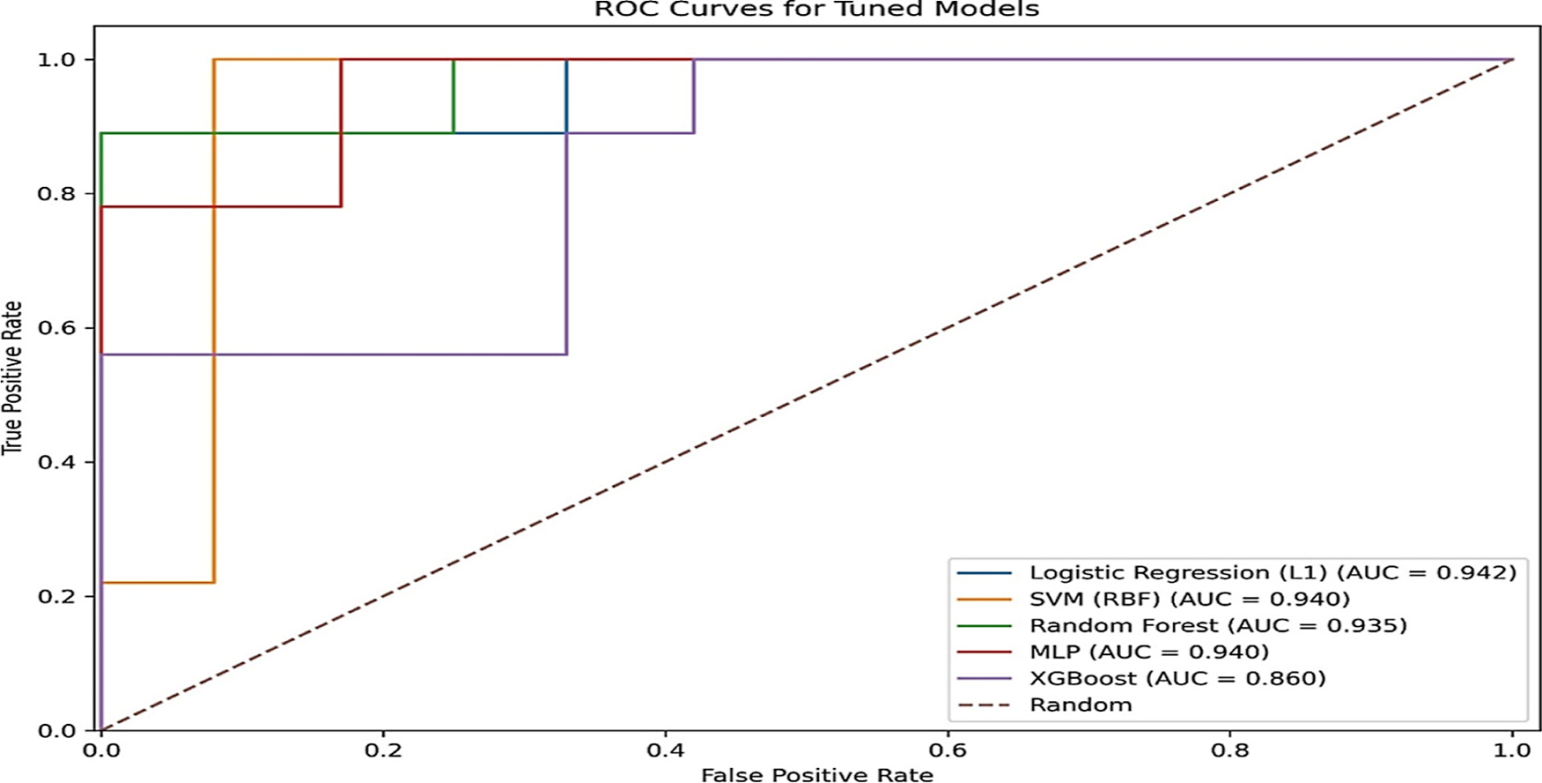
ROC curves for tuned logistic regression, SVM, random forest, MLP, and gradient boosting classifiers.

**Table 5:** Cross-Validated Performance (Metabolites Only; Stratified 5-fold; Mean ± SD)

model	accuracy (mean ± SD)	AUC (mean ± SD)
logistic regression (L1)	0.902 ± 0.061	0.963 ± 0.032
SVM (RBF kernel)	0.780 ± 0.095	0.900 ± 0.087
random forest	0.826 ± 0.086	0.926 ± 0.063
MLP (1 × 100)	0.805 ± 0.097	0.903 ± 0.075
XGBoost	0.765 ± 0.105	0.851 ± 0.088

Feature attribution supports biological coherence rather than model idiosyncrasy. In the binned spectral representation, LR-L1 yielded a sparse solution emphasizing 1.18 and 3.70 ppm (higher in BC) and 1.50 ppm (higher in controls), with additional contributions near 1.74 and 1.06 ppm. Random forest importance similarly prioritized 1.18, 3.70, 3.66, 1.34, and 1.50 ppm. These positions align with OPLS-DA/VIP assignments (e.g., ∼1.18 ppm ≈ 3-hydroxybutyrate; ∼3.70 ppm ≈ glucose), indicating ketone body utilization, glucose handling, and amino acid metabolism. Taken together, the machine learning results corroborate chemometric separation, provide threshold-free discrimination metrics for algorithm comparison, and yield interpretable feature-level contributions that map to NMR assignments and biochemical pathways. These findings are based only on internal validation and should therefore be interpreted cautiously.

To investigate the added value of clinical covariates in conjunction with metabolites, we conducted an exploratory hypothesis-generating analysis by using internal cross-validation. After exporting the data set and integrating the clinical variables, 101 samples (44 BC and 57 HC) were analyzed. Features were autoscaled, and a filter retained 30 variables for classification (methods). Cross-validation suggested that models incorporating clinical covariates also exhibited discriminatory potential across the same five classifiers. However, because this analysis was exploratory, internally validated, and performed in a modest sample, these estimates should be interpreted with caution. The SHAP attribution ranked carnitine, myoinositol, malonate, and 3-hydroxybutyrate as the most influential features, in agreement with chemometric findings. Independent external validation is required before clinical inferences can be drawn.

As a sensitivity analysis approximating the predictive subspace of OPLS-DA, we evaluated a PLS-DA pipeline in which foldwise PLS scores (four components) served as inputs to a logistic regression classifier under stratified 5-fold cross-validation. The mean test accuracy was 0.851 with an AUROC of 0.915. A 200-label permutation test yielded a p-value of 0.005, indicating that performance was unlikely to occur by chance (Supporting Information Table S3 and Supporting Information Figure S3).

Feature attribution supports biological coherence rather than model idiosyncrasy. In the binned spectral representation, LR-L1 yielded a sparse solution emphasizing 1.18 and 3.70 ppm (higher in BC) and 1.50 ppm (higher in controls), with additional contributions near 1.74 and 1.06 ppm, respectively. The random forest importance similarly prioritized 1.18, 3.70, 3.66, 1.34, and 1.50 ppm. These positions align with OPLS-DA/VIP assignments (e.g., ∼1.18 ppm ≈ 3-hydroxybutyrate; ∼3.70 ppm ≈ glucose), indicating ketone body utilization, glucose handling, and amino acid metabolism. Taken together, the machine learning results corroborate chemometric separation, provide threshold-free discrimination metrics suitable for algorithm comparison, and yield interpretable feature-level contributions that map to NMR assignments and biochemical pathways. Exploratory cross-validated analysis incorporating clinical covariates suggested additional discriminatory signals but should be considered hypothesis-generating. Because these analyses are based only on internal validation, external independent validation is required before clinical application.

As a sensitivity analysis approximating the predictive subspace of OPLS-DA, we evaluated a PLS-DA pipeline in which foldwise PLS scores (four components) served as inputs to a logistic regression classifier under stratified 5-fold cross-validation. The mean test accuracy was 0.851 with an AUROC of 0.915. A 200-label permutation test yielded a p-value of 0.005, indicating that performance was unlikely to occur by chance (Supporting Information Table S3 and Supporting Information Figure S3). The confusion matrices for the tuned machine learning models indicated a relatively balanced sensitivity and specificity for LR-L1, with weaker classification performance for RF, MLP, and XGBoost (Supporting Information Figure S5).

Figure 6 shows the ROC curves for the tuned logistic regression (L1), SVM-RBF, random forest, MLP, and XGBoost models evaluated by using an internal held-out test partition (20% of the cohort, *n* = 20). The hyperparameters were optimized within the training set by using cross-validation. The legend reports the corresponding AUC values, and the dashed diagonal denotes chance performance.

### 3.5 Pathway Enrichment and Differential Metabolite Analyses

To place the serum alterations in a biochemical context, 39 annotated metabolites were analyzed using MetaboAnalyst human KEGG pathways (metabolite set enrichment and pathway topology). Several pathways were significantly perturbed in the BC group compared to the HC group (Supporting Information Table S7; α = 0.05). The most enriched pathway was glyoxylate and dicarboxylate metabolism (raw *p* = 3.60 × 10^–7^; FDR = 1.81 × 10^–5^), which involved seven metabolites (e.g., glycine, formate, and citrate). Glycine, serine, and threonine metabolism was also highly significant (raw *p* = 4.52 × 10^–7^; seven metabolites, including glycine, serine, and threonine), as was alanine, aspartate, and glutamate metabolism (*p* = 3.24 × 10^–6^; six metabolites). Pathways linked to central carbon metabolism also showed evidence of disruption, including butanoate metabolism (*p* ≈ 7.06 × 10^–5^) and the citrate (tricarboxylic acid [TCA]) cycle (*p* ≈ 3.78 × 10^–3^). Additional changes were observed in branched-chain amino acid biosynthesis (valine, leucine, and isoleucine; *p* = 2.12 × 10^–4^), arginine and proline metabolism, and nitrogen metabolism. The pathway impact values were generally modest, indicating broad, distributed shifts rather than the alteration of a single pathway. Overall, this pattern implies dysregulation of energy production and amino acid utilization in BC serum (Figure 7; Supporting Information Table S7).

**Figure 7:**
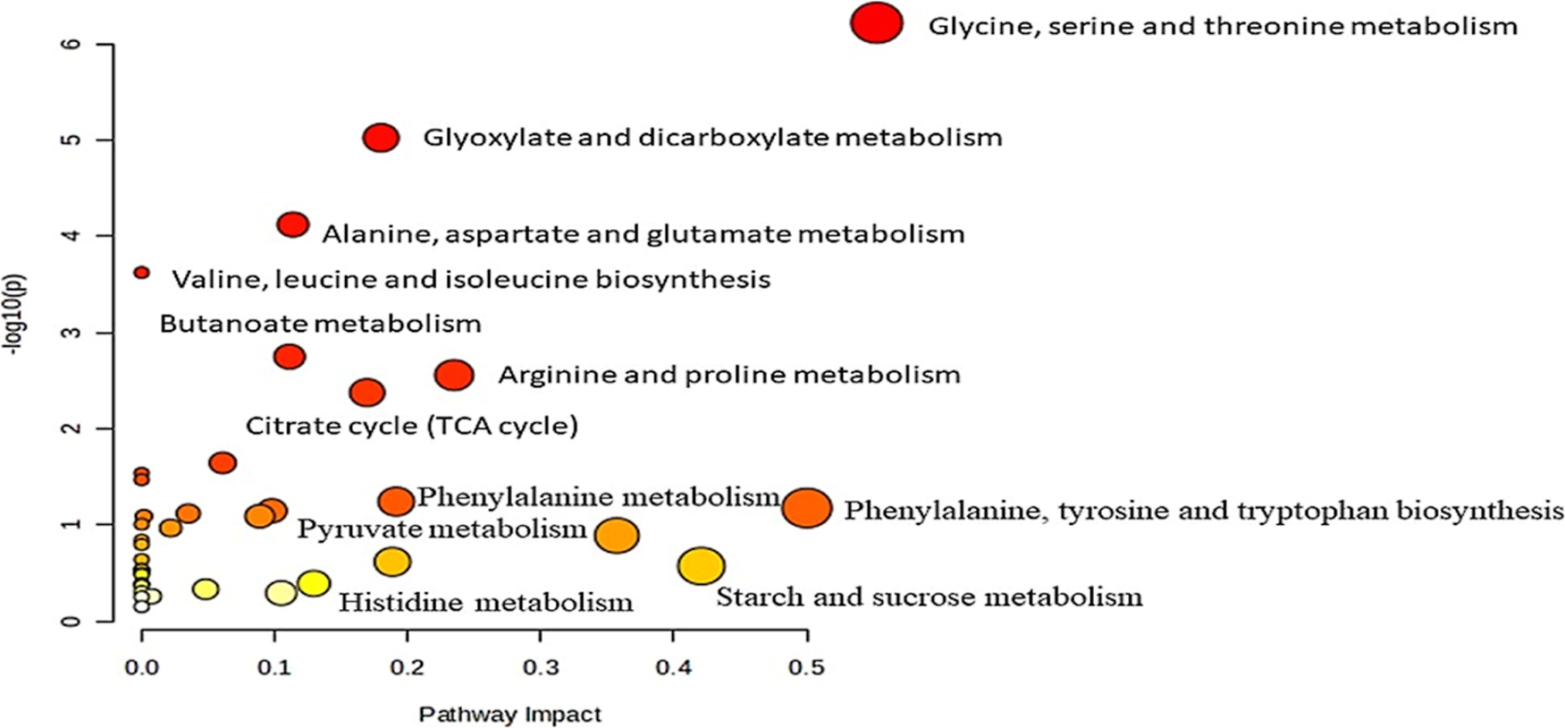
Pathway enrichment and topology analysis (KEGG).

Complementing the multivariate results, univariate analysis was used to compare each metabolite between the groups (volcano plot, Figure 8). Fourteen metabolites met the significance criterion (*p* <0.05, after multiple-test adjustment): eight were higher in BC (3-hydroxybutyrate, malonate, L-phenylalanine, dimethyl sulfone, citrate, myo-inositol, glutamine, and L-carnitine; all fold-changes >1), and six were lower (formate, L-tyrosine, histamine, an overlapping signal annotated as “Histamine1” [putative *p*-hydroxyphenylacetate], acetylsalicylate, and “Acetylsalicylate1” [salicylate]). Of these 14 metabolites, acetylsalicylate and salicylate were classified as exogenous pharmaceutical markers (aspirin and its metabolite) rather than endogenous cancer biomarkers (see Discussion); thus, the endogenous discriminant panel comprised 12 metabolites. Approximate effect sizes for selected metabolites were as follows: 3-hydroxybutyrate (FC ∼2.0, *p* ∼10^–7^), malonate (∼4.5, *p* ∼10^–7^), phenylalanine (∼1.8, *p* ∼10^–5^), dimethyl sulfone (∼1.7, *p* ∼10^–5^), citrate (∼2.3, *p* ∼10^–4^), myo-inositol (∼1.5, *p* ∼10^–3^), glutamine (∼1.3, *p* ∼10^–3^), carnitine (∼1.4, *p* ∼0.01), and, on the down-regulated side, formate (∼0.5, *p* ∼10^–5^), tyrosine (∼0.6, *p* ∼10^–3^), histamine (∼0.7, *p* ∼0.002), and acetylsalicylate (∼0.7, *p* ∼0.004). These findings are consistent with the OPLS-DA/VIP analyses, which highlighted ketone body utilization, glycolysis/glucose handling, and amino acid metabolism as the principal axes of discrimination.

**Figure 8:**
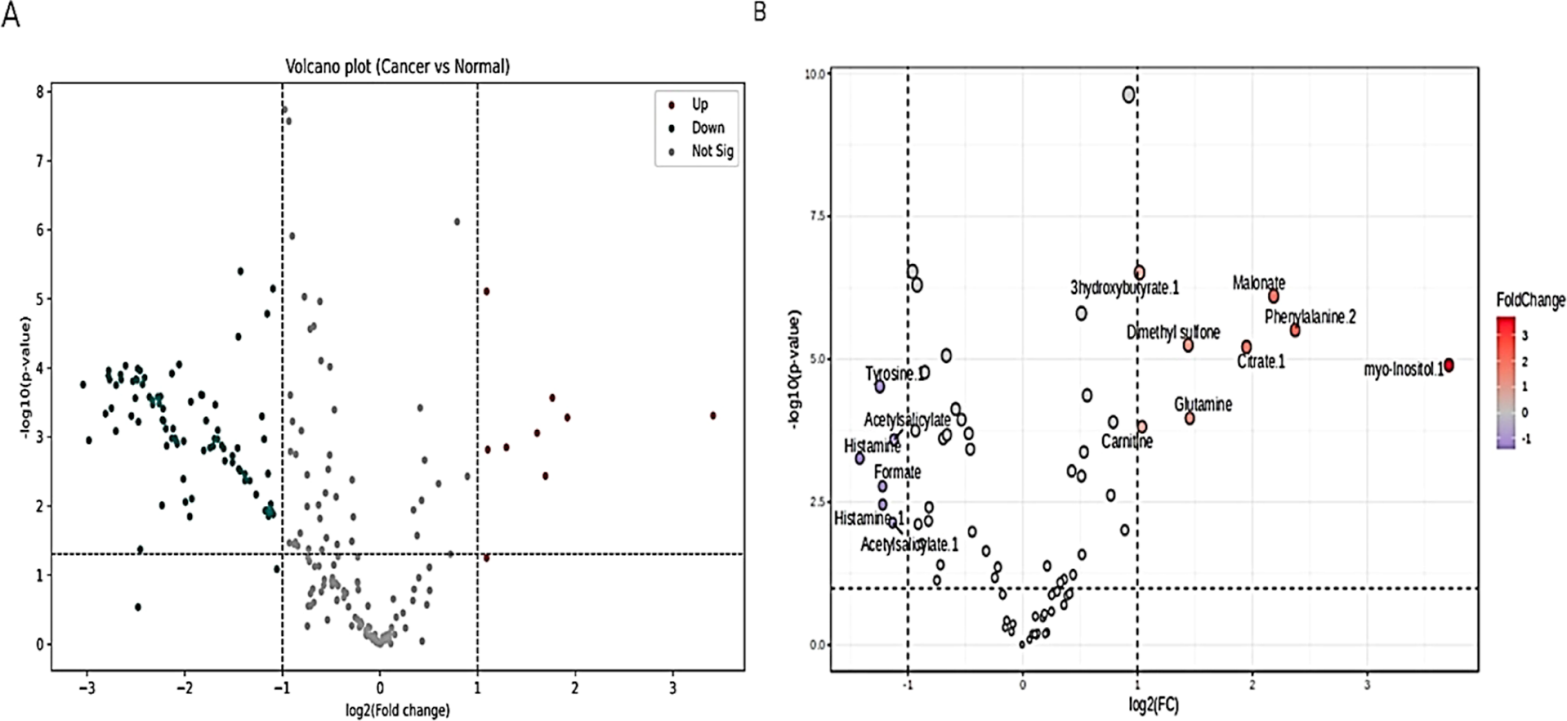
Volcano plot (BC vs HC).

The groupwise distributions of the 14 significant metabolites are shown in Figure 9. Consistent with the volcano plot, BC samples exhibited higher median levels and limited interquartile overlap for 3-hydroxybutyrate, malonate, and phenylalanine than those of HC samples. In contrast, the levels of formate, tyrosine, and histamine were lower in BC than in HC. A pathway network integrating the significant metabolites (Figure 10) highlighted coordinated changes across connected pathways, with hubs around TCA intermediates (e.g., citrate and succinate) and amino acid nodes (glycine, serine, and glutamate), mirroring the enrichment analysis and indicating system-level reprogramming rather than isolated metabolite shifts.

**Figure 9:**
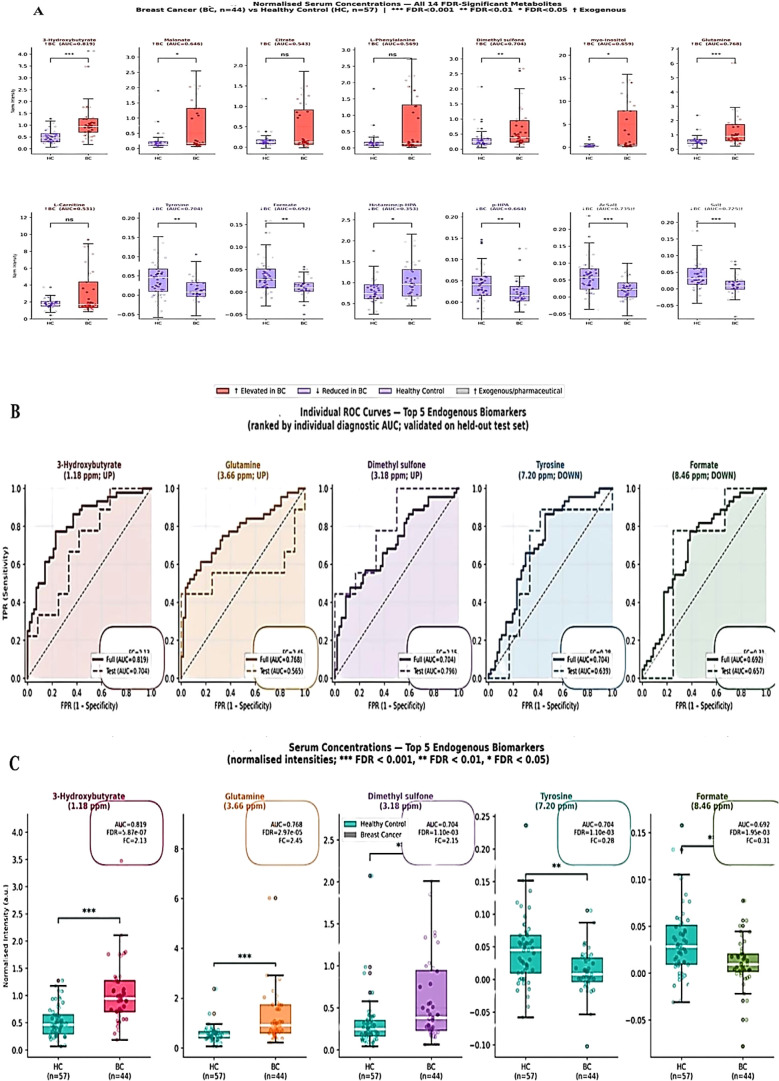
Serum metabolic alterations associated with breast cancer.

**Figure 10:**
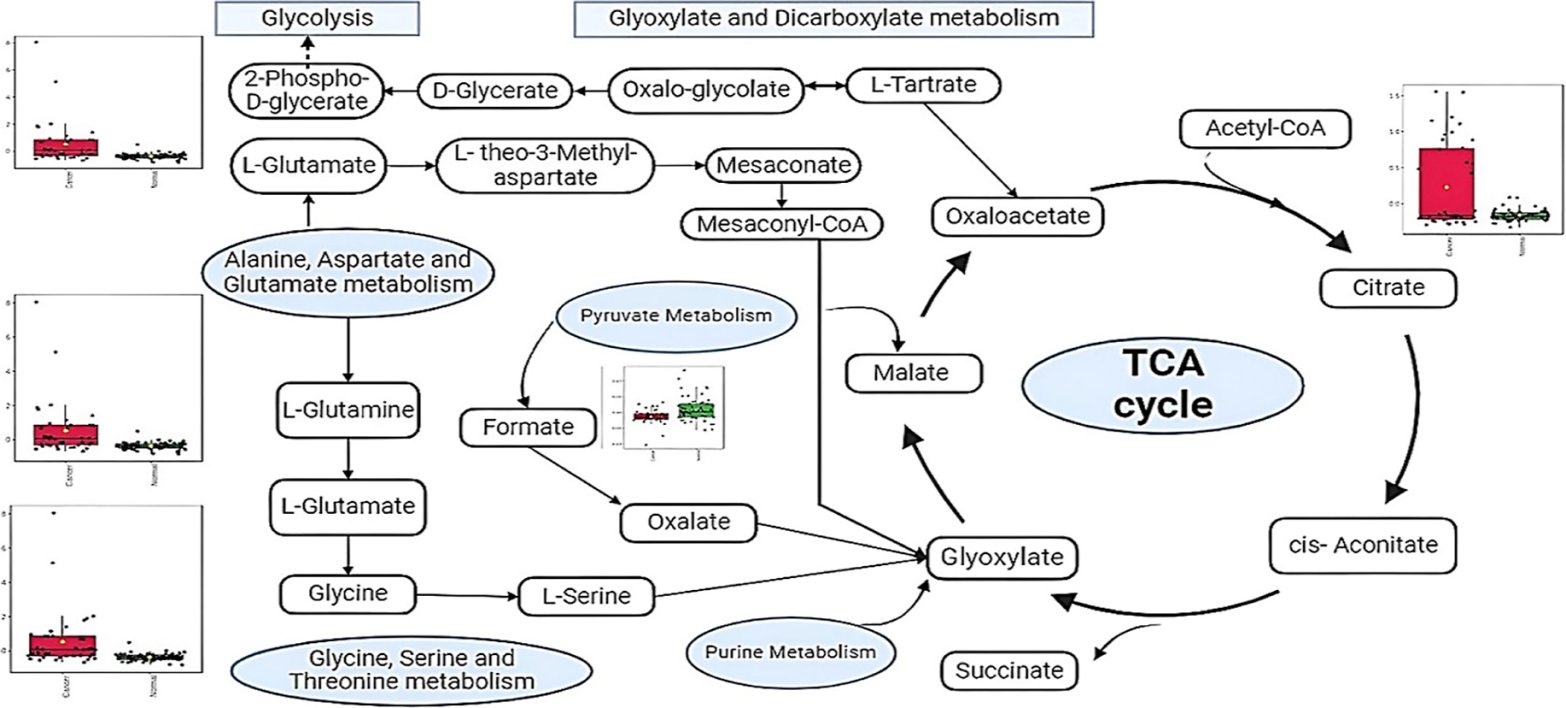
Integrative pathway map of altered serum metabolites in breast cancer.

Bubble plot from MetaboAnalyst was used for 39 discriminant metabolites. Color encodes –log_10_(p) (white to red = increasing significance), and size encodes the pathway impact. Significant pathways (FDR-adjusted *p* ≤0.05) included glyoxylate and dicarboxylate metabolism (raw *p* = 3.60 × 10^–7^; FDR = 1.81 × 10^–5^), glycine/serine/threonine metabolism (raw *p* = 4.52 × 10^–7^), alanine/aspartate/glutamate metabolism (*p* = 3.24 × 10^–6^), butanoate metabolism (*p* ≈ 7.06 × 10^–5^), branched-chain amino acid biosynthesis (*p* = 2.12 × 10^–4^), and the citrate (TCA) cycle (*p* ≈ 3.78 × 10^–3^). The complete statistics are provided in Supporting Information Table S7.

Figure 8A,B shows each point represents a metabolite; the *x*-axis is log_2_(fold-change) (positive = higher in BC), and the *y*-axis is–log_10_(p). The horizontal dashed line marks the significance threshold (adjusted *p* <0.05), and the vertical dashed lines mark |log_2_FC| = 1.

Among the 14 metabolites that remained significant after false discovery rate correction, eight were significantly elevated in patients with breast cancer compared to healthy controls, whereas six were significantly reduced. Elevated metabolites included 3-hydroxybutyrate, malonate, citrate, l-phenylalanine, dimethyl sulfone, myo-inositol, glutamine, and l-carnitine, whereas reduced metabolites included formate, tyrosine, histamine, *p*-hydroxyphenylacetate, acetylsalicylate, and salicylate. Acetylsalicylate and salicylate were identified as exogenous aspirin-related signals and were therefore excluded from downstream modeling, leaving 12 endogenous discriminatory metabolites. Receiver operating characteristic analysis of the five highest-ranked endogenous metabolites (3-hydroxybutyrate, glutamine, dimethyl sulfone, tyrosine, and formate), performed on a held-out test set, produced AUC values of 0.692–0.819, with FDR-corrected fold changes ranging from 0.28 to 2.45. These results indicate a consistent serum metabolic signature associated with breast cancer and suggest perturbations in ketone body metabolism, amino acid metabolism, methylation-associated intermediates, and one-carbon metabolism, which may be relevant for biomarker development (Figure 9).

Normalized serum metabolite concentrations were compared between patients with breast cancer (BC, *n* = 44) and healthy controls (HCs, *n* = 57) for all metabolites that remained significant after false discovery rate (FDR) correction. (A) Among the 14 FDR-significant metabolites, eight were elevated in BC (3-hydroxybutyrate, malonate, citrate, L-phenylalanine, dimethyl sulfone, myo-inositol, glutamine, and L-carnitine), whereas six were reduced (formate, tyrosine, histamine, *p*-hydroxyphenylacetate, acetylsalicylate, and salicylate). Acetylsalicylate and salicylate were identified as exogenous aspirin-related metabolites and were therefore excluded from the final 12-metabolite endogenous discriminatory panel. (B) ROC analysis of the five highest-ranked endogenous metabolites (3-hydroxybutyrate, glutamine, dimethyl sulfone, tyrosine, and formate) yielded AUC values of 0.692–0.819 in the held-out test set. (C) Individual metabolite distributions for these five candidates further illustrated significant differences between BC and HC, with FDR-corrected p-values and fold changes ranging from 0.28 to 2.45. In the box plots, the center line represents the median, boxes indicate the interquartile range, whiskers extend to 1.5 × IQR, and individual observations are shown. Significance is denoted as ***FDR <0.001, **FDR <0.01, and *FDR <0.05. † indicates an exogenous/pharmaceutical marker.

Based on the metabolite-level differences shown in Figure 9, we examined whether the altered metabolites converged into specific biochemical pathways. Pathway mapping indicated that significant endogenous metabolites were functionally connected within networks related to glycolysis/pyruvate metabolism, glyoxylate and dicarboxylate metabolism, alanine/aspartate/glutamate metabolism, glycine/serine/threonine metabolism, and the TCA cycle. This integrative view supports the interpretation that breast cancer is associated with the coordinated reprogramming of central energy and amino acid metabolism rather than isolated changes in individual metabolites (Figure 10).

Differential serum metabolites were positioned within interconnected metabolic pathways to provide a biochemical context for the serum alterations observed in breast cancer. Metabolites are shown as rounded rectangles, pathway modules as blue ovals, and arrows indicate known biochemical relationships. The insets summarize the distributions of the selected metabolites in the breast cancer and the healthy control groups. The network illustrates coordinated alterations linking glycolysis, glyoxylate and dicarboxylate metabolism, alanine/aspartate/glutamate metabolism, pyruvate metabolism, glycine/serine/threonine metabolism, purine metabolism, and the tricarboxylic acid (TCA) cycle. The corresponding pathway assignments and statistics are presented in Supporting Information Table S7.

## 4 Discussion

This ^1^H NMR metabolomics study profiled fasting serum samples from Malaysian women with breast cancer (BC) and healthy controls (HCs) by integrating chemometrics and supervised machine learning to investigate disease-associated metabolic alterations. These findings demonstrate a distinct serum metabolomic profile in patients with BC, reflecting systematic metabolomic reprogramming. OPLS-DA showed clear group separation, whereas supervised machine learning achieved a strong diagnostic performance.

However, these results should be interpreted cautiously because validation was performed internally and has not yet been confirmed in an independent external cohort. Previous metabolomics studies using complementary platforms, such as GC–MS, have also reported high diagnostic performance in external validation cohorts, supporting the broader translational potential of circulating metabolic biomarkers for noninvasive cancer detection.^29^

A total of 14 serum metabolites were significantly dysregulated in patients with BC. Eight metabolites were elevated, including 3-hydroxybutyrate, citrate, glutamine, malonate, phenylalanine, dimethyl sulfone (DMSO_2_), myo-inositol, and l-carnitine, whereas six were reduced, including formate, tyrosine, histamine, *p*-hydroxyphenylacetate, acetylsalicylate, and salicylate. Acetylsalicylate and salicylate were likely exogenous pharmaceutical markers of aspirin exposure rather than endogenous cancer-specific biomarkers and were therefore excluded from the endogenous biomarker panel. The remaining 12 metabolites indicated the major axes of breast-cancer-associated metabolic dysregulation, including altered energy metabolism, amino acid metabolism, and broader host metabolic responses.

Elevated 3-hydroxybutyrate levels suggest increased fatty acid β-oxidation and ketogenesis, consistent with the ability of cancer cells to exploit ketone bodies as oxidative fuels under selected metabolic conditions.^30^ The concomitant elevation of L-carnitine levels further supports enhanced lipid mobilization and mitochondrial fatty acid transport, indicating metabolomic flexibility in the use of fatty-derived fuels.^31^ TCA cycle-related perturbations, particularly increased citrate and malonate levels, were also observed. Citrate accumulation may reflect altered oxidative utilization or diversion toward biosynthetic pathways, whereas malonate, a competitive inhibitor of succinate dehydrogenase, may indicate partial suppression of TCA cycle flux and an adaptive response to oxidative stress.^32^ These findings are supported by the pathway enrichment results, which highlighted the glyoxylate and dicarboxylate metabolism, TCA/citrate cycle, ketone body metabolism, and amino acid pathways.

Amino acid metabolism is another major discriminatory axis. Phenylalanine increased, whereas tyrosine decreased, resulting in an elevated phenylalanine/tyrosine ratio. This pattern is compatible with inflammation-associated metabolic dysregulation, in which pro-inflammatory cytokines may reduce phenylalanine hydroxylase activity and contribute to phenylalanine accumulation.^33^ Lower histamine and *p*-hydroxyphenylacetate levels may also reflect altered immune-, microbiome-, or medication-associated metabolism, although these findings should be interpreted with caution. Glutamine is modestly elevated despite its known consumption by proliferating tumor cells for anaplerosis and nucleotide biosynthesis.^34,35^ This may reflect compensatory systemic mobilization of glutamine from host tissues to meet tumor-associated metabolic demand.

A comparison with previous studies suggests both shared and population-specific metabolic features. For example, a Spanish ^1^H NMR metabolomics study reported higher levels of branched-chain amino acids and tyrosine in BC patients,^7^ whereas tyrosine was reduced in the present Malaysian cohort. Such differences may reflect variations in the genetic background, dietary patterns, menopausal status, tumor subtype, analytical workflow, or population-specific metabolic profiles. Therefore, this study contributes important preliminary data from a Southeast Asian cohort and supports the need for population-specific biomarker reference data, as signatures identified in Western populations may not be translated directly into Asian cohorts. Several additional metabolites can provide relevant biological and contextual insights. Increased DMSO_2_ may reflect oxidative stress, sulfur metabolism, gut microbial activity, or dietary and supplement exposure.^36^ Higher myo-inositol levels may be related to altered membrane turnover or osmoprotective responses. In contrast, reduced acetylsalicylate and salicylate signals most likely reflect differences in aspirin or NSAID exposure rather than BC biology.^37^ This distinction highlights the importance of separating endogenous metabolic alterations from exogenous pharmaceutical- or lifestyle-related signals when defining translational biomarker panels for BC.

Therefore, the potential effects of lipid-related clinical variables should be considered. Total cholesterol, triglyceride, ApoB100, and FABP4 levels differed between the groups and may reflect a broader host metabolic status. To reduce macromolecule and lipoprotein contributions, spectra were acquired using the CPMG spin–echo sequence, and sensitivity analysis excluding characteristic lipoprotein-associated spectral bins produced minimal changes in classifier performance. This suggests that group discrimination is not primarily driven by lipoprotein-dominated spectral regions. Nevertheless, future studies should incorporate detailed fasting lipid profiles, adipokine measurements, and covariate-adjusted analyses with larger external validation cohorts.

From a translational perspective, the present findings support a five-metabolite endogenous panel comprising 3-hydroxybutyrate, glutamine, dimethyl sulfone, tyrosine, and formate. These metabolites represent complementary biological processes, including energy reprogramming, oxidative-stress-related sulfur metabolism, aromatic amino acid imbalance, and one-carbon metabolism. A reduced three-metabolite panel comprising 3-hydroxybutyrate, glutamine, and dimethyl sulfone may also be useful for targeted ^1^H NMR or biosensor-based assays. However, these panels should only be considered candidate signatures and require independent prospective validation before clinical application.

Pathway and network analyses further support coordinated system-level reprogramming rather than isolated metabolite changes. Enrichment pathways included central carbon metabolism, the TCA cycle, ketone body metabolism, glyoxylate/dicarboxylate pathways, and multiple amino acid pathways. Alterations in formate and glycine are also consistent with one-carbon metabolic activity, which is closely linked to proliferation metabolism.^38^ Although specific metabolite changes vary across studies, a broad pathway-level overlap is evident across different populations and analytical platforms, reinforcing the biological plausibility of identified metabolic alterations.

This study has several strengths, including a fasting, treatment-naïve case–control design, age-matched healthy controls, and exclusion of major comorbidities such as diabetes, thereby reducing noncancer sources of metabolic variation. However, this study has several limitations that must be acknowledged. The analyzed cohort comprised 101 participants after spectral quality control exclusion, and all samples were obtained from a single Malaysian center. Therefore, the held-out test set was an internal subset rather than a geographically or temporally independent validation cohort. The cross-sectional design also limits the assessment of longitudinal metabolic trajectories or prediagnostic predictive value. Additional limitations include the limited power of subtype-specific analyses and possible residual confounding from menopausal status, diet, microbiome variation, medication use, and lipid-related clinical variables.

Finally, since ^1^H NMR spectroscopy preferentially detects mid-to-high-abundance polar metabolites, its integration with complementary MS-based platforms, including lipidomics and targeted steroid/eicosanoid assays, may broaden the pathway coverage and improve the biomarker sensitivity. A planned multicenter prospective cohort incorporating complete medication histories, matched lifestyle data, multiplatform profiling, and independent external validation is essential to confirm the diagnostic performance and assess the clinical utility.

In summary, this study provides proof-of-concept evidence that ^1^H NMR-based serum metabolomics combined with advanced machine learning can distinguish BC patients from healthy controls in a Malaysian cohort. The identified metabolic alterations involved energy metabolism, amino acid pathways, one-carbon metabolism, and lipid-related fuel utilization. Although promising, the findings remain preliminary, because they were supported only by internal validation. Larger multicenter and multiethnic studies with independent external validation are required to confirm diagnostic performance, assess disease specificity, and establish clinical relevance. Nevertheless, these data support metabolic phenotyping as a promising strategy for developing a population-tailored approach to BC detection and risk stratification.

## 5 Conclusion

In a fasting, treatment-naïve Malaysian case–control cohort (*n* = 101 analyzed; 120 enrolled), untargeted ^1^H NMR serum metabolomics was combined with supervised learning distinguished women with breast cancer from healthy controls (OPLS-DA *R* ^2^Y = 0.851; *Q* ^2^ = 0.722; accuracy 90.1%; AUC = 0.942 on an internal held-out test partition). The discriminant profile, including higher levels of 3-hydroxybutyrate, citrate, glutamine, and phenylalanine and lower tyrosine and histamine levels, indicated coordinated disturbances in central carbon and energy metabolism, amino acid and one-carbon pathways, and lipid/fatty acid utilization, consistent with tumor-associated metabolic reprogramming. These findings support the further evaluation of serum metabolomics as a noninvasive adjunct to existing diagnostic pathways in Southeast Asian settings; however, independent multicenter validation is required to establish generalizability and clinical utility.

## Supplementary Material



## Data Availability

The data supporting this study are publicly available in Mendeley Data.^39^

## Notes

OPLS-DA permutation validation of the BC versus HC discrimination model (200 permutations, one predictive component); OPLS-DA permutation validation of the healthy control versus BC discrimination model (200 permutations, one predictive component); ROC curve and permutation test for the PLS-DA model coupled to logistic regression (stratified 5-fold CV); feature attribution for the corrected metabolite-only classification pipeline: signed L1-LR coefficients and Random Forest importance, confusion matrices for tuned classifiers (L1-LR, RF, SVM-RBF) on the internal held-out test partition (*n* = 20); CONSORT-style participant flow diagram for the serum ^1^H NMR breast cancer metabolomics study (*n* = 120 enrolled); ROC curves and repeated cross-validation performance of classification models on the internal held-out test set (n = 120 enrolled); repeated stratified 5-fold cross-validation performance (mean accuracy and AUC ± SD) for L1-LR, SVM-RBF, RF, MLP, and GBM classifiers; CV-ANOVA results for the OPLS-DA model (one predictive component); variable importance in projection scores for the top 33 OPLS-DA discriminant spectral bins; cross-validated performance of the four component PLS-DA model coupled to logistic regression (stratified 5-fold CV); tuned hyperparameters for the metabolite-only ML classifiers (L1-LR, RF, SVM-RBF); comparative cross-validated ML classifier performance for BC versus HC discrimination; top discriminating spectral bins identified by L1-penalized logistic regression and Random Forest feature attribution; pathway enrichment analysis results for BC-associated metabolites; differential metabolite summary statistics for the 14 significantly altered metabolites; sensitivity analysis after exclusion of lipoprotein-associated spectral bins; and sensitivity analysis of classifier performance after exclusion of exogenous metabolite bins (acetylsalicylate and salicylate) (PDF)
