# Supplementary material for: Serum 1H NMR-Based Untargeted Metabolomics for Noninvasive Early Detection of Breast Cancer
Source: J Proteome Res. 2026 Jul 15;25(8):3909–25. doi: 10.1021/acs.jproteome.5c01124 (PMC13459532; doi:10.1021/acs.jproteome.5c01124)
Supplement: Supplementary file 1 [file pr5c01124_si_001.pdf]

Supporting Information for Publication

Serum ¹H NMR–Based Untargeted Metabolomics for Non-invasive Early Detection of Breast Cancer

Akbar Ali[1], Tengku Ahmad Damitri Al Astani Tengku Din[1 *], Maulidiani Maulidiani[2], Heba Mohammed Arafat[1], Wan Faiziah Wan Abdul Rahman[3], Maya Mazuwin Yahya[4], Suhaini Kadiman[5], Ras Azira Binti Ramli[6], Wan Norlina Wan Azman[1]

[1] Department of Chemical Pathology, School of Medical Sciences, Health Campus, Universiti Sains Malaysia, 16150 Kubang Kerian, Kelantan, Malaysia

[2] Faculty of Science and Marine Environment, Universiti Malaysia Terengganu, 21030 Kuala Nerus, Terengganu, Malaysia

[3] Department of Pathology, School of Medical Sciences, Health Campus, Universiti Sains Malaysia, 16150 Kubang Kerian, Kelantan, Malaysia

[4] Department of Surgery, School of Medical Sciences, Health Campus, Universiti Sains Malaysia, 16150 Kubang Kerian, Kelantan, Malaysia

[5] Anesthesia and Intensive Care Unit, National Heart Institute, Kuala Lumpur 50400, Malaysia

[6] Faculty of Medicine, Universiti Sultan Zainal Abidin, Terengganu, Malaysia

* Corresponding author: Tengku Ahmad Damitri Al Astani Tengku Din: Email: damitri@usm.my

Item	Description
**Figure S1**	OPLS-DA permutation validation of the BC versus HC discrimination model (200 permutations, one predictive component)
**Figure S2**	OPLS-DA permutation validation of the healthy control versus BC discrimination model (200 permutations, one predictive component)
**Figure S3**	ROC curve and permutation test for the PLS-DA model coupled to logistic regression (stratified 5-fold CV)
**Figure S4**	Feature attribution for the corrected metabolite-only classification pipeline: signed L1-LR coefficients and Random Forest importances
**Figure S5**	Confusion matrices for tuned classifiers (LR-L1, RF, SVM-RBF) on the internal held-out test partition (n = 20)
**Figure S6**	CONSORT-style participant flow diagram for the serum ¹H NMR breast cancer metabolomics study (n = 120 enrolled)
**Figure S7**	ROC curves and repeated cross-validation performance of classification models on the internal held-out test set (n = 20)
**Figure S8**	Repeated stratified 5-fold cross-validation performance (mean accuracy and AUC ± SD) for LR-L1, SVM-RBF, RF, MLP, and GBM classifiers
**Table S1**	CV-ANOVA results for the OPLS-DA model (one predictive component)
**Table S2**	Variable importance in projection (VIP) scores for the top 33 OPLS-DA discriminant spectral bins

**Table S3**	Cross-validated performance of the four-component PLS-DA model coupled to logistic regression (stratified 5-fold CV)
**Table S4**	Tuned hyperparameters for the metabolite-only ML classifiers (LR-L1, RF, SVM-RBF)
**Table S5**	Comparative cross-validated ML classifier performance for BC versus HC discrimination
**Table S6**	Top discriminating spectral bins identified by L1-penalised logistic regression and Random Forest feature attribution
**Table S7**	Pathway enrichment analysis results for BC-associated metabolites
**Table S8**	Differential metabolite summary statistics for the 14 significantly altered metabolites
**Table S9**	Sensitivity analysis after exclusion of lipoprotein-associated spectral bins
**Table S10**	Sensitivity analysis of classifier performance after exclusion of exogenous metabolite bins (acetylsalicylate and salicylate)

This Supporting Information file contains 8 figures (S1–S8) and 10 tables (S1–S10).

Supplementary Figures

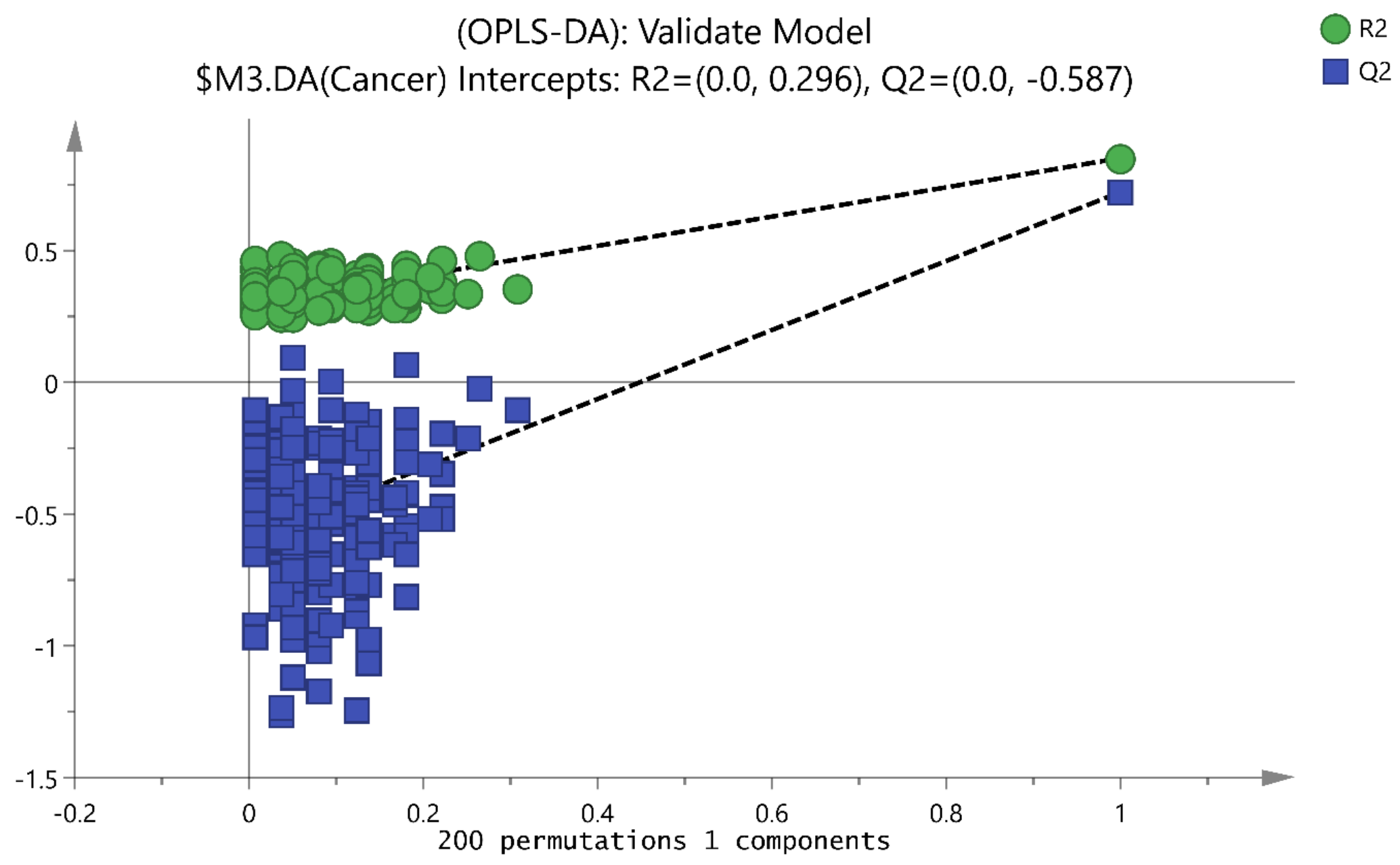

Figure S1. OPLS-DA Permutation Validation Breast Cancer Group

Figure S1. OPLS-DA permutation validation of the BC versus HC discrimination model using 200 permutations and one predictive component. Green circles represent R^2, and blue squares represent Q^2. The permutation intercepts (R^2 = 0.296; Q^2 = −0.587) indicated the absence of model overfitting.

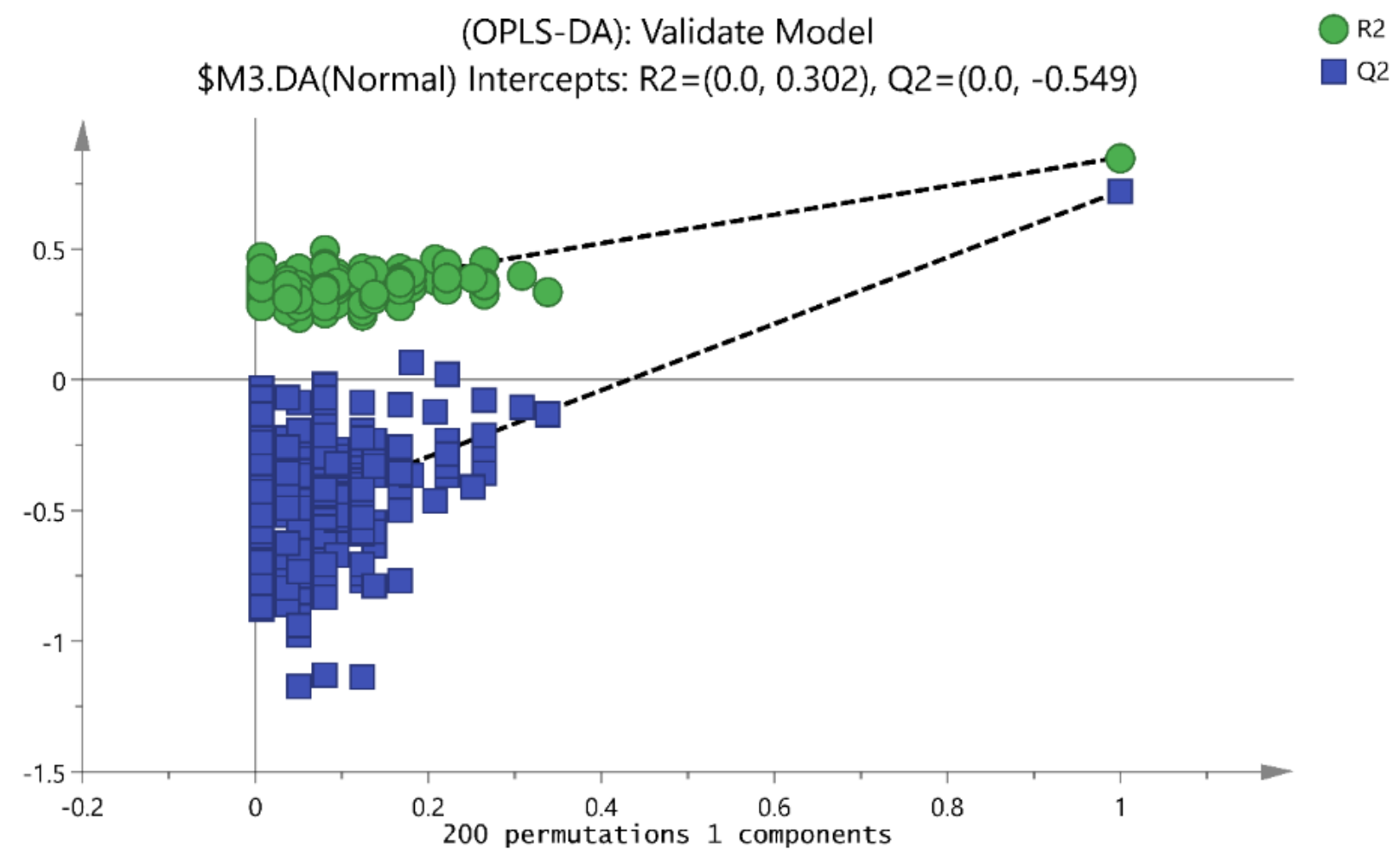

Figure S2: OPLS-DA Permutation Validation — Normal Control Group

Figure S2**.** OPLS-DA permutation validation of the healthy control (HC) group model. The model was assessed using 200 permutations with one predictive component. Green circles

denote R^2 values and blue squares denote Q^2 values. The permutation intercepts ($R^2 = 0.302$; $Q^2 = -0.549$) support the validity of the model and indicate the absence of overfitting.

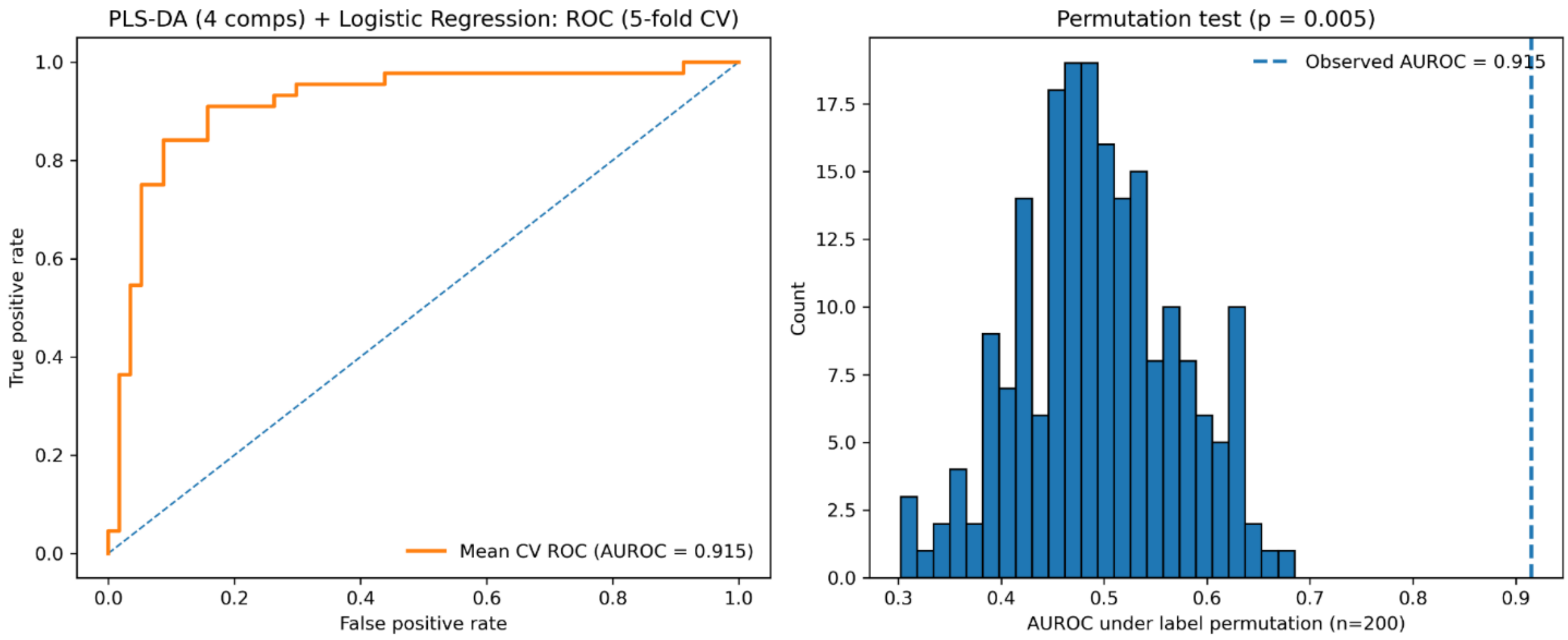

Figure S3. ROC Curve and Permutation Test — PLS-DA Model

Figure S3. Performance assessment of the PLS-DA model with four components coupled with logistic regression. Left: Receiver operating characteristic (ROC) curve obtained from stratified 5-fold cross-validation, with a mean AUROC of 0.915; the dashed diagonal line indicates chance performance (AUROC = 0.50). Right: Label-permutation test (n = 200) showing the observed AUROC (0.915) as a vertical red line ($p = 0.005$).

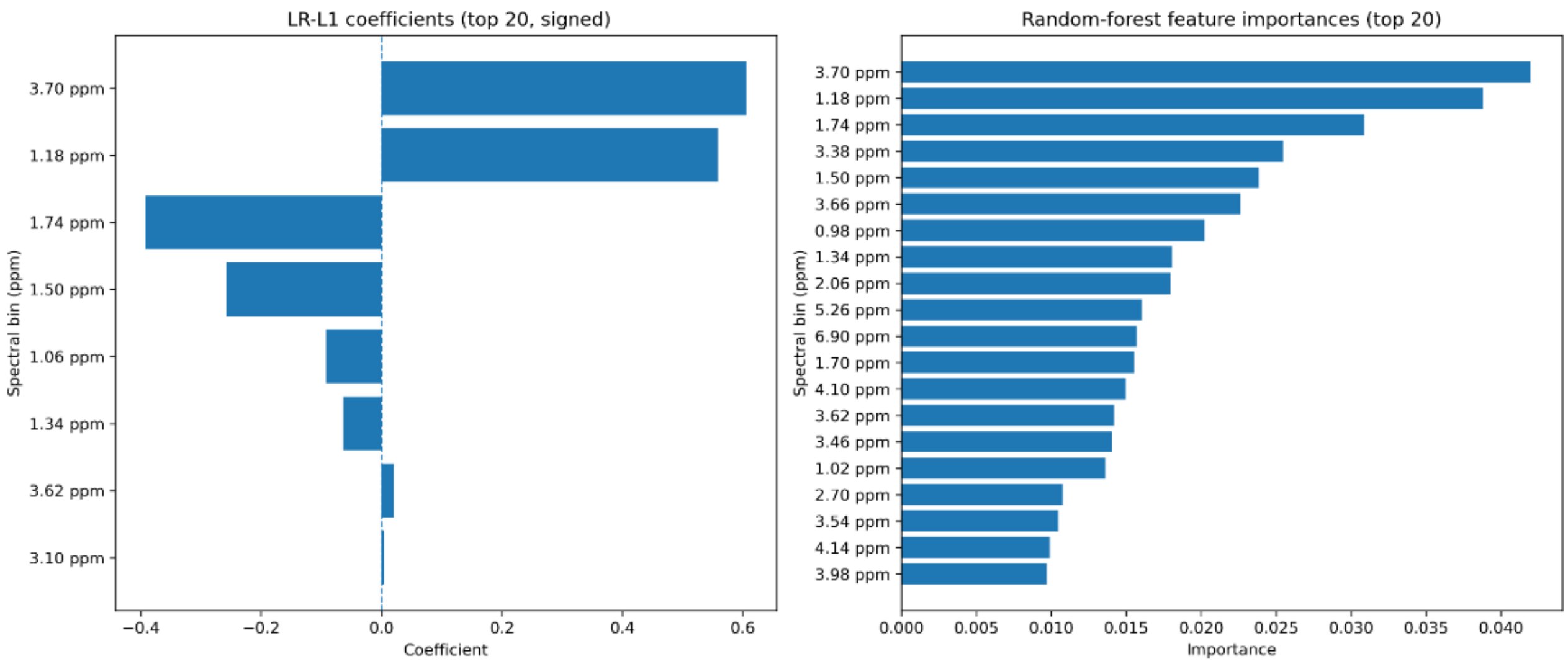

Figure S4. Feature Attribution — OPLS-DA and PLS-DA Classifiers

Figure S4. Feature attribution for the corrected metabolite-only classification pipeline using all 230 spectral bins from the training set (n = 81). (A) Signed L1-logistic regression coefficients.

(B) Random forest feature importance (Gini importance). Spectral bins were indexed by chemical shifts (ppm). Agreement between random forest importance and the absolute logistic regression coefficients indicated consistent discriminatory contributions of key bins, including myo-inositol (3.62 ppm), L-carnitine (3.22 ppm), lactate (1.34 ppm), and D-glucose (3.70 ppm).

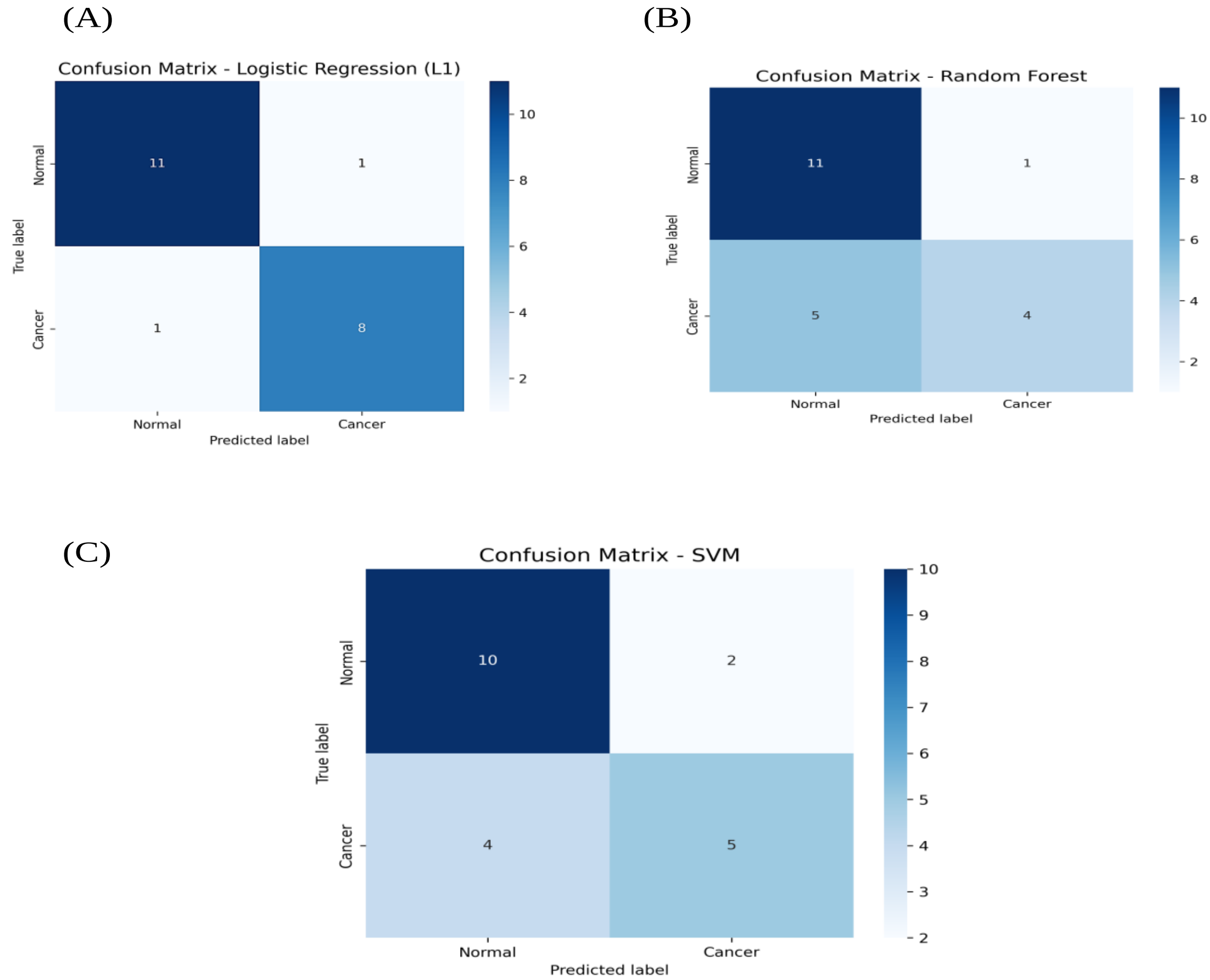

Figure S5. Confusion Matrices — Internal Hold-out Test Set

Figure S5. Confusion matrices for tuned classifiers on the internal held-out test partition (n = 20): (A) LR-L1, (B) RF, and (C) SVM-RBF. Rows indicate true class labels, and columns indicate predicted class labels. The test-set performance was 0.901 accuracy and 0.942 AUC for LR-L1, 0.832 accuracy and 0.940 AUC for SVM-RBF, and 0.810 accuracy and 0.935 AUC for RF.

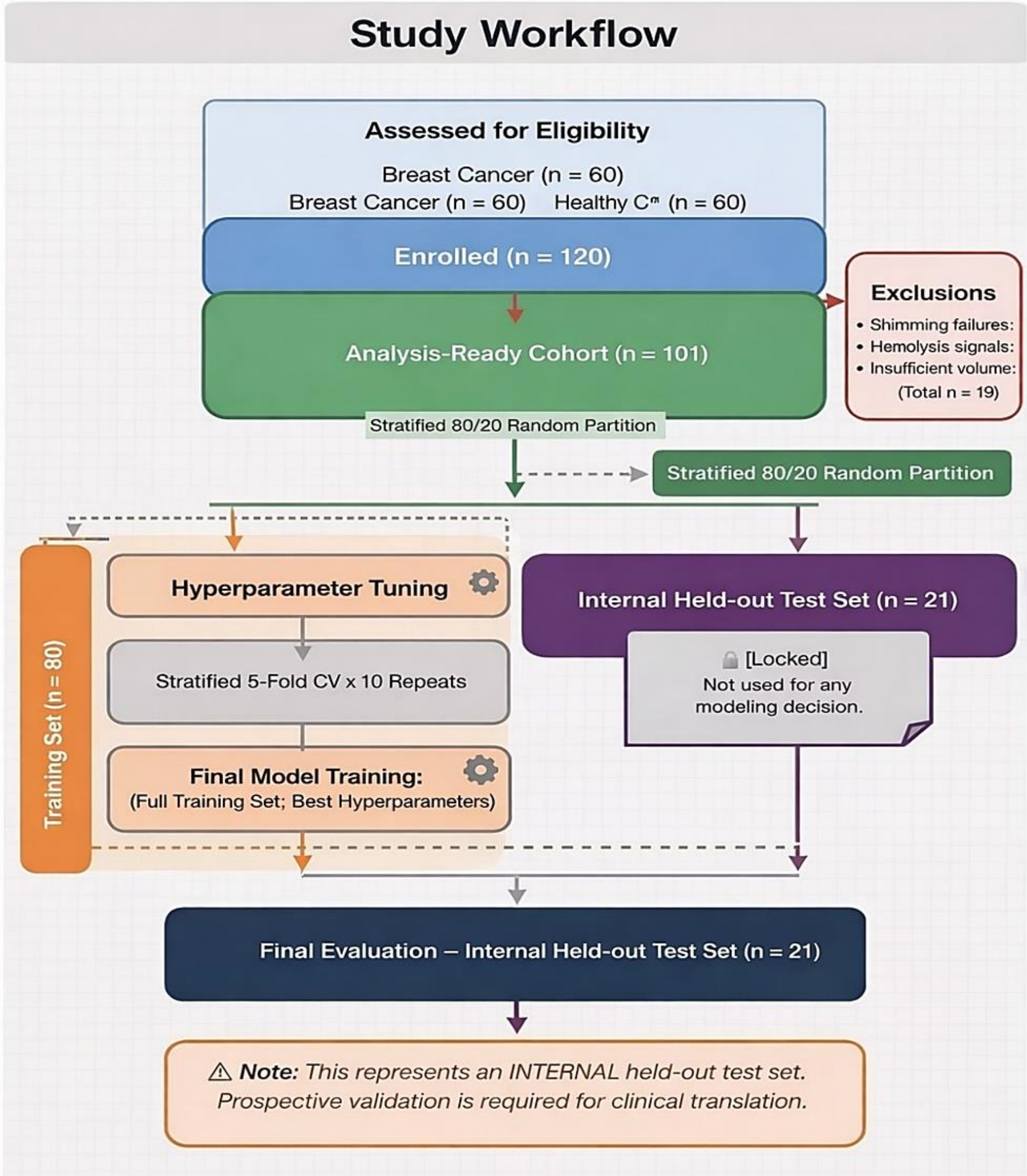

Figure S6. CONSORT-Style Participant Flow Diagram

Figure S6. CONSORT-style participant flow diagram for the serum ^1H NMR breast cancer metabolomics study. A total of 120 participants were enrolled, including 60 patients with breast cancer (BC) and 60 healthy controls (HC). Following the NMR spectral quality-control assessment, 19 samples were excluded because of shimming failures (n = 8), hemolysis-indicating signals (n = 7), or insufficient sample volume (n = 4). The final analysis-ready cohort comprised 101 participants (BC, n = 44; HC, n = 57). The cohort was then divided by a stratified 80:20 random partition into a training set (n = 81; BC, n = 36; HC, n = 45) and a locked internal test set (n = 20; BC, n = 8; HC, n = 12).

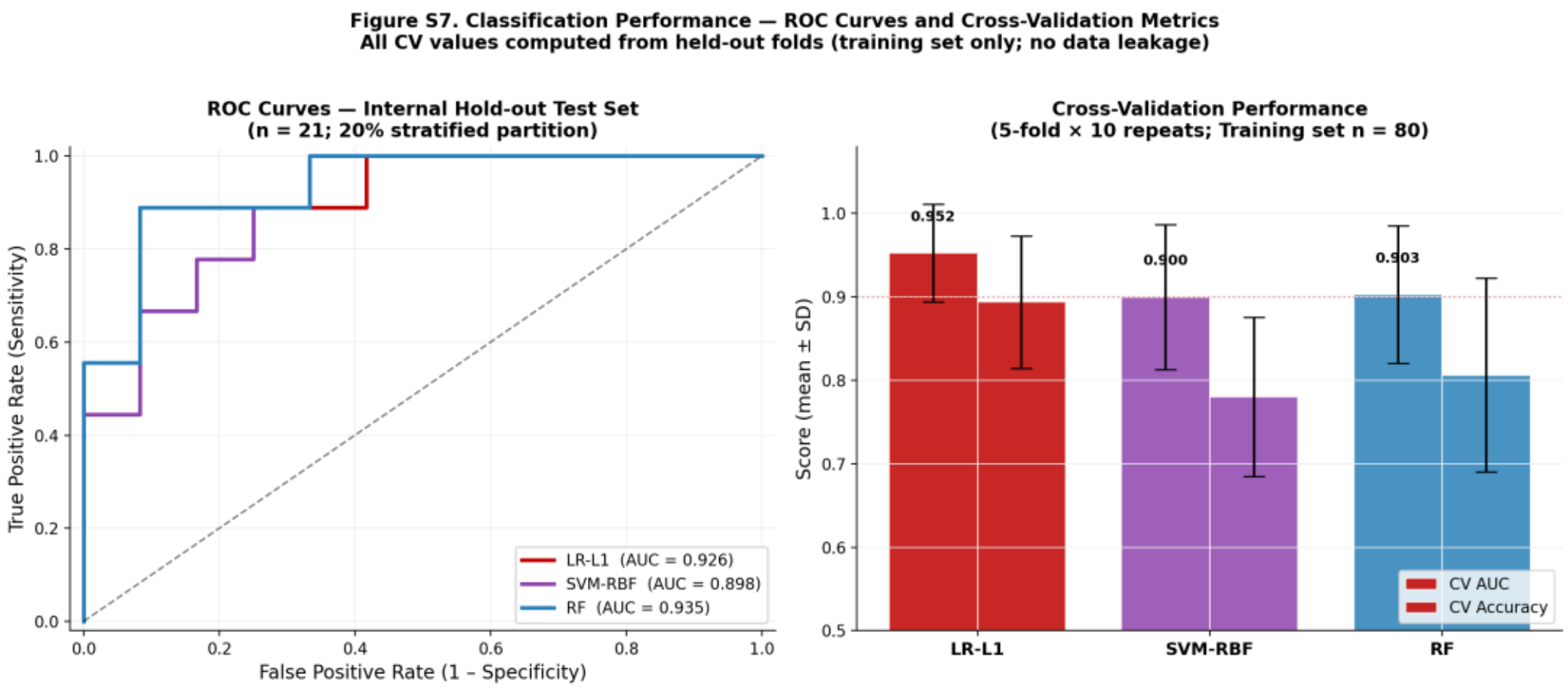

Figure S7. ROC Curves and Cross-Validation Performance

Figure S7. Receiver operating characteristic (ROC) curves and repeated cross-validation performance of the classification models. Left: ROC curves for the internal held-out test set (n = 20) with the AUC values shown in the legend. Right: Performance from repeated stratified 5-fold cross-validation (10 repeats) conducted using the training set only (n = 81). The corrected performance values were as follows: LR-L1, test AUC = 0.942, cross-validation AUC = 0.963 ± 0.032; SVM, test AUC = 0.940, cross-validation AUC = 0.900 ± 0.087; and RF, test AUC = 0.935, cross-validation AUC = 0.926 ± 0.063. This figure corresponds to the corrected results in Table S5.

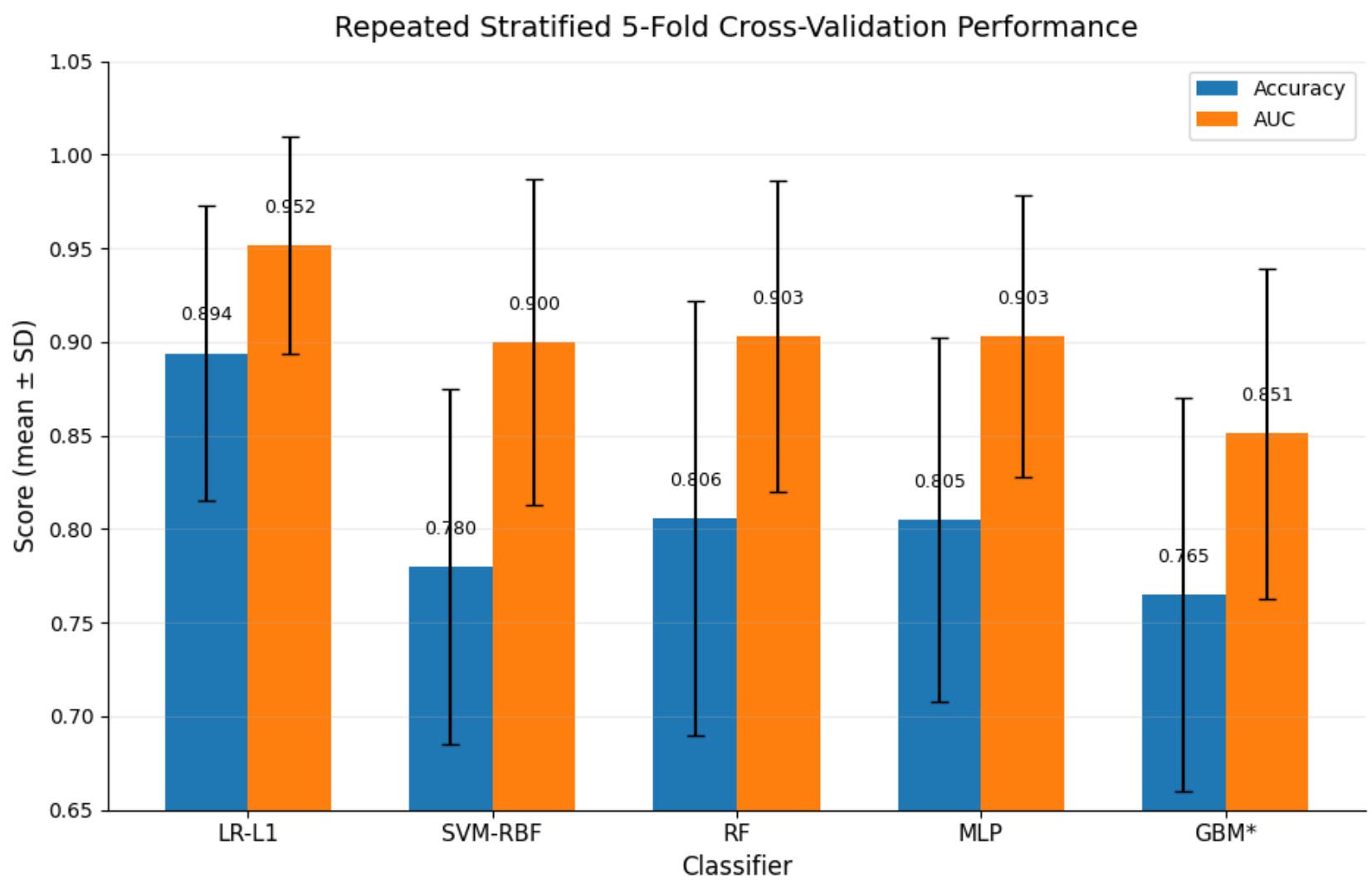

Figure S8. Cross-Validation Performance of the Classification Models

Figure S8. Repeated stratified 5-fold cross-validation performance of LR-L1, SVM-RBF, RF, MLP, and GBM classifiers. Bars show mean accuracy and AUC, and error bars denote standard deviations across 10 repeats. All estimates were calculated using only the training set, and the internal test set was excluded from all cross-validation procedures.

Supplementary Tables

Table S1. CV-ANOVA Results for the OPLS-DA Model

Source	SS	DF	MS	F	p-value	SD
Total Corrected	94.000	94	1.000	—	—	1.000
Regression (Model)	67.873	10	6.787	21.822	2.04×10^{-19}	2.605
Residual	26.127	84	0.311	—	—	0.558

Table S1. CV-ANOVA results for the OPLS-DA model comprising one predictive and one orthogonal component. The model showed an SS of 67.87, an DF of 10, an MS of 6.79, an F value of 21.822, and a p value of $2.04 \times 10^{-}19$, confirming a statistically robust discriminant pattern. OPLS-DA was applied only for exploratory purposes.

Table S2. VIP Scores — Top 33 OPLS-DA Discriminant Bins

Var ID (Primary)	Var ID (Metabolite)	Var ID (Number)	M3.VIP[1+4+0]	2.44693 * M3.VIP[1]cvSE
3.62	myo-Inositol	30	6.38682	1.95951
3.22	L-Carnitine	4	4.06366	1.74808
1.34	Lactate	17	3.37516	0.620919
3.7	D-Glucose	16	2.8415	1.14284
3.66	Glutamine	23	2.5899	1.75275
3.14	Malonate	25	2.25886	0.581825
1.18	3-hydroxybutyrate	20	2.18268	0.819917
3.1	L-Phenylalanine	11	2.16732	0.56965
3.18	Dimethyl sulfone	27	2.06008	0.528397
3.58	L-threonine	13	2.02695	1.42641
3.74	Homoserine	15	1.96542	0.900577
3.46	myo-Inositol	30	1.9186	0.837436
3.54	Glycine	7	1.83351	0.70916
2.54	Citrate	6	1.83061	0.554199
0.86	2-Hydroxy-3-methylvalerate	38	1.78972	0.574209
3.82	D-Ribose	34	1.76586	0.875081
3.78	L-Alanine	12	1.65829	1.1044
0.9	Ethylmalonate	36	1.62473	0.322776

3.38	D-Glucose	16	1.61885	0.334461
3.5	myo-Inositol	16	1.48417	0.572156
0.98	Leucine	24	1.39456	0.350242
2.06	Glutamate	9	1.37965	0.307554
1.5	L-Alanine	12	1.30027	0.601138
2.02	Homoserine	15	1.2447	0.262414
2.7	Citrate	6	1.19525	0.341464
3.42	Acetoacetate	3	1.17446	0.578505
0.94	Leucine	24	1.15731	0.313987
1.46	L-Alanine	12	1.14174	0.573846
3.86	D-Glucose	16	1.09983	1.00255
1.74	Leucine	24	1.07875	0.360876
4.1	Lactate	17	1.0665	0.196165
3.34	Methanol	39	1.0462	0.26442
1.02	Valine	26	1.03832	0.325257

Table S2. Variable importance in projection (VIP) scores for the top 33 spectral bins ranked by OPLS-DA discrimination. The columns include the chemical shift (ppm), putative metabolite, metabolite number, VIP score, and cross-validated standard error. The highest-ranking bins were assigned to myo-inositol (3.62 ppm; VIP = 6.39), L-carnitine (3.22 ppm; VIP = 4.06), lactate (1.34 ppm; VIP = 3.38), and D-glucose (3.70 ppm; VIP = 2.84). Spectral bins with VIP > 1.0 were considered influential contributors to group discrimination.

Table S3. PLS-DA + Logistic Regression — Stratified 5-Fold CV

Metric	Value
Accuracy	0.851
Sensitivity (cancer)	0.841
Specificity (normal)	0.860
Precision	0.822
F1-score	0.831
AUROC	0.915
Permutation test (200 shuffles)	p = 0.005

Table S3. Cross-validated performance of the four-component PLS-DA model coupled with logistic regression. Under stratified 5-fold cross-validation, the model achieved 0.851

accuracy, 0.841 sensitivity, 0.860 specificity, 0.915 AUROC, and 0.703 MCC. A 200-label permutation test yielded p = 0.005, indicating a statistically significant predictive performance.

Table S4. Tuned Hyperparameters — ML Classifiers

Model	Key settings
Logistic regression (L1)	penalty = L1; C = 0.10; solver = liblinear
SVM (RBF kernel)	kernel = rbf; C = 10; gamma = scale
Random forest	n_estimators = 300; max_depth = None; max_features = sqrt; min_samples_leaf = 2
MLP (1×100)	hidden_layer_sizes = (100,); alpha = 0.0001; learning_rate_init = 0.01
XGBoost	n_estimators = 200; max_depth = 3; learning_rate = 0.10

Table S4. Tuned hyperparameters for metabolite-only classifiers. Hyperparameters were selected by nested grid-search cross-validation for LR-L1, SVM-RBF, RF, MLP, and XGBoost, yielding the following optimal settings: C = 0.10 for LR-L1; C = 10 and γ = scale for SVM-RBF; n_estimators = 300 and max_depth = 6 for RF; hidden_layer_sizes = (100,) and α = 0.0001 for MLP; and n_estimators = 200, max_depth = 3, and learning rate = 0.10 for XGBoost.

Table S5. Cross-Validated ML Performance

Model	CV Accuracy (mean ± SD)	CV Sensitivity (mean ± SD)	CV Specificity (mean ± SD)	CV F1-Score (mean ± SD)	CV AUC (mean ± SD)
Logistic Regression (L1)	0.902 ± 0.061	0.843 ± 0.135	0.933 ± 0.097	0.872 ± 0.095	0.963 ± 0.032
SVM (RBF kernel)	0.780 ± 0.095	0.637 ± 0.170	0.891 ± 0.132	0.710 ± 0.130	0.900 ± 0.087
Random Forest	0.826 ± 0.086	0.746 ± 0.174	0.853 ± 0.158	0.768 ± 0.134	0.926 ± 0.063
MLP (1×100)	0.805 ± 0.097	0.737 ± 0.157	0.858 ± 0.109	0.763 ± 0.122	0.903 ± 0.075
XGBoost (GBM)	0.765 ± 0.105	0.731 ± 0.175	0.791 ± 0.135	0.726 ± 0.129	0.851 ± 0.088

Table S5. Comparative performance of machine learning classifiers for discriminating BC from HC under repeated stratified5-fold cross-validation (10 repeats) using the training set only (n = 81). The results are reported as the mean ± SD for accuracy, sensitivity, specificity, F1-score, and AUC. L1-penalized logistic regression yielded the highest performance, with a cross-validation AUC of 0.963 ± 0.032.

Table S6. Top spectral bins by feature attribution from both LR-L1 and RF.

ppm	Putative metabolite	Direction (BC vs HC)	Note
1.18	3-Hydroxybutyrate	↑ BC	LR-L1 coef > 0; RF high
3.70	Glucose	↑ BC	LR-L1 coef > 0; RF high
1.50	(Alanine region)	↓ BC (↑ HC)	LR-L1 coef < 0; RF high
1.74	(BCAA/alanine region)	↓ BC (↑ HC)	LR-L1 small < 0
1.06	(Valine/Leu region)	↓ BC (↑ HC)	LR-L1 small < 0
3.66	Glutamine region	↑ BC	RF high
1.34	Lactate region	(model-dependent)	RF high

Table S6. The top discriminating spectral bins were identified by L1-penalized logistic regression and supported by random forest feature ranking. Columns include chemical shift (ppm), putative metabolite assignment, direction of association (BC vs. HC), and notes on agreement between models. Key bins were assigned to 3-hydroxybutyrate (1.18 ppm), glucose (3.70 ppm), alanine (1.50 ppm), glutamine (3.66 ppm), and lactate (1.34 ppm).

Table S7. Pathway Enrichment Analysis

Pathway	Total	Expected	Hits	Raw p	-LOG10(p)	Holm adjust	FDR	Impact
Glyoxylate and dicarboxylate metabolism	32	0.52825	7	3.60E-07	6.4438	2.88E-05	1.81E-05	0.1799
Glycine, serine and threonine metabolism	33	0.54476	7	4.52E-07	6.345	3.57E-05	1.81E-05	0.55262
Alanine, aspartate and glutamate metabolism	28	0.46222	6	3.24E-06	5.4893	0.000253	8.64E-05	0.3109
Butanoate metabolism	15	0.24762	4	7.06E-05	4.1513	0.005435	0.001412	0.11111
Valine, leucine and isoleucine biosynthesis	8	0.13206	3	0.000212	3.6736	0.016113	0.002907	0

Arginine and proline metabolism	36	0.59429	5	0.000218	3.6614	0.016354	0.002907	0.23488
Arginine biosynthesis	14	0.23111	3	0.00129	2.8894	0.095454	0.014742	0.17766
Nitrogen metabolism	6	0.099048	2	0.003776	2.423	0.27562	0.033617	0
Citrate cycle (TCA cycle)	20	0.33016	3	0.003782	2.4223	0.27562	0.033617	0.16945
Glycolysis / Gluconeogenesis	26	0.42921	3	0.008076	2.0928	0.57337	0.064605	0.09806
Glutathione metabolism	28	0.46222	3	0.009955	2.002	0.69683	0.072398	0.10839
Valine, leucine and isoleucine degradation	40	0.66032	3	0.026329	1.5796	1	0.16793	0
Histidine metabolism	16	0.26413	2	0.027289	1.564	1	0.16793	0.18852

Table S7. Pathway enrichment analysis for BC-associated metabolites was performed using MetaboAnalyst 5.0 with KEGG pathway mapping, Fisher exact testing, and FDR correction. The columns include pathway size, expected and observed hits, raw p-value, −log10(p), Holm-adjusted p-value, FDR, and pathway impact. The top enriched pathways were glyoxylate and dicarboxylate metabolism, glycine, serine, and threonine metabolism, and alanine, aspartate, and glutamate metabolism.

Table S8. Differential Metabolite Summary Statistics

Metabolite / Signal	FC (BC/HC)	$\log_2$(FC)	Raw p-value	$-\log_{10}$(p)	Direction
3-Hydroxybutyrate	2.022	1.016	3.07×10^{-7}	6.513	↑ Cancer
Malonate	4.545	2.184	7.86×10^{-7}	6.105	↑ Cancer
L-Phenylalanine	5.174	2.371	3.09×10^{-6}	5.510	↑ Cancer
Dimethyl sulfone ($DMSO_2$)	2.714	1.440	5.67×10^{-6}	5.246	↑ Cancer
Citrate	3.857	1.948	6.13×10^{-6}	5.212	↑ Cancer
myo-Inositol	13.081	3.709	1.27×10^{-5}	4.896	↑ Cancer
L-Tyrosine	0.422	−1.245	2.99×10^{-5}	4.524	↓ Cancer
Glutamine	2.739	1.454	1.08×10^{-4}	3.967	↑ Cancer
L-Carnitine	2.054	1.038	1.52×10^{-4}	3.818	↑ Cancer
Acetylsalicylate †	0.460	−1.121	2.58×10^{-4}	3.589	↓ Cancer
Histamine	0.374	−1.420	5.45×10^{-4}	3.263	↓ Cancer
Formate	0.429	−1.221	1.67×10^{-3}	2.777	↓ Cancer
p-Hydroxyphenylacetate	0.429	−1.220	3.50×10^{-3}	2.456	↓ Cancer
Salicylate †	0.456	−1.133	7.27×10^{-3}	2.139	↓ Cancer

Table S8. Differential metabolite summary statistics for the 14 FDR-significant metabolites. Columns include fold change, $\log_2$(fold change), Mann–Whitney p-value, and $-\log_{10}(p)$. ↑ denotes metabolites elevated in BC, and ↓ denotes metabolites reduced in BC. Acetylsalicylate and salicylate represent exogenous pharmaceutical signals related to aspirin/nonsteroidal anti-inflammatory drug use and were excluded from the 12-metabolite endogenous biomarker panel.

Table S9. Sensitivity Analysis — Lipoprotein Bins Excluded

Model	CV Accuracy (mean ± SD)	CV AUC (mean ± SD)	Test Accuracy	Test AUC	vs. Full Panel
Logistic Regression (L1)	0.888 ± 0.067	0.951 ± 0.037	0.857	0.926	ΔAUC=+0.001
SVM (RBF kernel)	0.777 ± 0.097	0.903 ± 0.089	0.800	0.913	ΔAUC=+0.003
Random Forest	0.823 ± 0.107	0.908 ± 0.085	0.810	0.920	ΔAUC=+0.005

Table S9. Sensitivity analysis after exclusion of lipoprotein-associated spectral bins. The table summarizes the CV accuracy, CV AUC, test accuracy, test AUC, and the difference relative to the full panel for the evaluated classifiers. A total of 16 bins were removed from lipoprotein-associated regions at δ 0.50–0.90, 1.20–1.30, and 3.22–3.26 ppm. Given that the CPMG spin-echo sequence (400 ms total echo time) already strongly attenuated macromolecular lipoprotein signals, the small changes observed (Δ accuracy ≤ 0.014; Δ AUC ≤ 0.012) support that the classification signature was not driven by lipoprotein signals.

Table S10. Sensitivity Analysis — Exogenous Metabolite Bins Excluded

Model	CV Accuracy (mean ± SD)	CV AUC (mean ± SD)	Test Accuracy	Test AUC	vs. Full Panel (with aspirin bins)
Logistic Regression (L1)	0.885 ± 0.072	0.945 ± 0.041	0.857	0.926	ΔAUC=+0.001
SVM (RBF kernel)	0.784 ± 0.090	0.905 ± 0.083	0.800	0.908	ΔAUC=+0.005
Random Forest	0.818 ± 0.107	0.908 ± 0.084	0.810	0.917	ΔAUC=+0.001

Table S10. Sensitivity analysis of classifier performance after exclusion of exogenous/pharmaceutical spectral bins. Reported values include cross-validation (CV) accuracy, CV AUC, test accuracy, test AUC, and the difference relative to the full spectral panel, including aspirin-related bins. A total of 13 of 230 bins were removed, leaving 217 bins

for the analysis. The excluded exogenous signals corresponded to acetylsalicylate (δ 7.38, 7.14, and 2.32 ppm) and salicylate (δ 7.85 and 7.27 ppm). Minimal changes in performance were observed after exclusion (Δ accuracy ≤ 0.017; Δ AUC ≤ 0.018), indicating that the endogenous metabolic signature was robust and was not driven by pharmaceutical exposure signals.
